# *Cyclospora cayetanensis* comprises at least 3 species that cause human cyclosporiasis

**DOI:** 10.1017/S003118202200172X

**Published:** 2023-03

**Authors:** Joel Leonard Nicholas Barratt, John Shen, Katelyn Houghton, Travis Richins, Sarah G. H. Sapp, Vitaliano Cama, Michael J. Arrowood, Anne Straily, Yvonne Qvarnstrom

**Affiliations:** 1 Parasitic Diseases Branch, Division of Parasitic Diseases and Malaria, Centers for Disease Control and Prevention, Atlanta, GA 30329, USA; 2 Epidemiology Department, The Rollins School of Public Health, Emory University, Atlanta, GA, USA; 3 Oak Ridge Institute for Science and Education, Oak Ridge Associated Universities, Oak Ridge, TN, USA; 4 Waterborne Disease Prevention Branch, National Center for Emerging and Zoonotic Infectious Diseases, Centers for Disease Control and Prevention, Atlanta, GA, USA

**Keywords:** *Cyclospora ashfordi*, *Cyclospora cayetanensis*, *Cyclospora henanensis*, epidemiology, genotyping, speciation

## Abstract

The apicomplexan parasite *Cyclospora cayetanensis* causes seasonal foodborne outbreaks of the gastrointestinal illness cyclosporiasis. Prior to the coronavirus disease-2019 pandemic, annually reported cases were increasing in the USA, leading the US Centers for Disease Control and Prevention to develop a genotyping tool to complement cyclosporiasis outbreak investigations. Thousands of US isolates and 1 from China (strain CHN_HEN01) were genotyped by Illumina amplicon sequencing, revealing 2 lineages (A and B). The allelic composition of isolates was examined at each locus. Two nuclear loci (CDS3 and 360i2) distinguished lineages A and B. CDS3 had 2 major alleles: 1 almost exclusive to lineage A and the other to lineage B. Six 360i2 alleles were observed – 2 exclusive to lineage A (alleles A1 and A2), 2 to lineage B (B1 and B2) and 1 (B4) was exclusive to CHN_HEN01 which shared allele B3 with lineage B. Examination of heterozygous genotypes revealed that mixtures of A- and B-type 360i2 alleles occurred rarely, suggesting a lack of gene flow between lineages. Phylogenetic analysis of loci from whole-genome shotgun sequences, mitochondrial and apicoplast genomes, revealed that CHN_HEN01 represents a distinct lineage (C). Retrospective examination of epidemiologic data revealed associations between lineage and the geographical distribution of US infections plus strong temporal associations. Given the multiple lines of evidence for speciation within human-infecting *Cyclospora*, we provide an updated taxonomic description of *C. cayetanensis*, and describe 2 novel species as aetiological agents of human cyclosporiasis: *Cyclospora ashfordi* sp. nov. and *Cyclospora henanensis* sp. nov. (Apicomplexa: Eimeriidae).

## Graphical abstract

**Figure:**
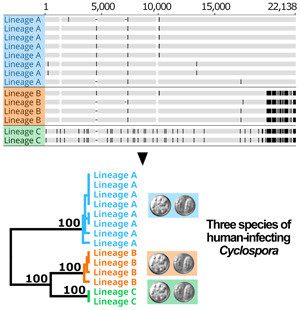

## Introduction

The foodborne apicomplexan parasite *Cyclospora cayetanensis* causes seasonal outbreaks of the diarrhoeal illness cyclosporiasis in the USA, with cases peaking typically from May to August (Casillas *et al*., 2018, 2019; Barratt *et al*., 2019; Nascimento *et al*., 2020). Prior to the coronavirus disease-2019 (COVID-19) pandemic, annually reported cyclosporiasis cases were increasing in the USA, leading the US Centers for Disease Control and Prevention (CDC) to develop a genotyping system to complement epidemiologic investigations of cyclosporiasis outbreaks (Barratt *et al*., 2019, 2021; Nascimento *et al*., 2020). Early iterations of this system were evaluated on isolates collected during the cyclosporiasis peak period of 2018 (Nascimento *et al*., 2020), and evaluations continued in 2019 and 2020 (Barratt *et al*., 2021, 2022). These evaluations supported the system's epidemiologic utility as isolates from epidemiologically linked case-patients were typically assigned to the same genetic cluster (Nascimento *et al*., 2020; Barratt *et al*., 2021, 2022). Consequently, CDC's *C. cayetanensis* genotyping system continues to be used to complement outbreak investigations, and a library of *C. cayetanensis* genotypes has been steadily expanding.

CDC's *C. cayetanensis* genotyping system (i.e. ‘CYbernetic CLustering Of Non-clonal Eukaryotes’ – the ‘CYCLONE’ suite of workflows and algorithms) currently involves polymerase chain reaction (PCR) amplification of 8 genetic markers, including 2 encoded in the mitochondrial (Mt) genome and 6 in the nuclear (Nu) genome. The Mt markers include the repetitive junction region (Mt junction) (Nascimento *et al*., 2019), and a second locus referred to as ‘MSR’ (Barratt *et al*., 2019; Nascimento *et al*., 2020). The Nu markers include CDS1, CDS2, CDS3 and CDS4 described by Houghton *et al*. (2020), plus 2 additional markers referred to as ‘360i2’ and ‘378’ (Barratt *et al*., 2019; Nascimento *et al*., 2020). Resultant amplicons are deep-sequenced on the Illumina Miseq platform and the reads are supplied to the CYCLONE bioinformatic workflow comprising various modules (Barratt *et al*., 2021). Module 1 defines the genotype of isolates by compiling a list of haplotypes detected at each marker for each isolate (Barratt *et al*., 2021). Module 2 computes pairwise genetic distances from these genotypes using an ensemble learning approach (Nascimento *et al*., 2020; Jacobson *et al*., 2022). These genetic distances are subsequently clustered for downstream analysis (Barratt *et al*., 2021). Unlike traditional phylogenetic methods where tree structures are based on nucleotide differences observed in a multiple sequence alignment, the genetic distance computation algorithms underpinning module 2 (i.e. Barratt's heuristic definition of genetic distances and Plucinski's Bayesian algorithm) consider all haplotypes detected, including multiple haplotypes detected at heterozygous loci (Barratt *et al*., 2021; Jacobson *et al*., 2022). Consequently, the presence of very rare and very common allelic combinations greatly influences resultant tree structures, highlighting potential disruptions in gene flow.

From March 2018 to October 2020, 3459 *C. cayetanensis* genotypes were sequenced at CDC from fecal specimens collected in North and Central America (i.e. the Americas, referred to as ‘American’ herein). This included specimens from case-patients infected in the USA and Canada, and persons infected while travelling to or living in Mexico and Guatemala. American specimens collected prior to 2018 (i.e. from 2014 to 2017) were also genotyped retrospectively. Genetic distances were computed from resultant genotypes, and subsequent clustering revealed 2 distinct populations. To increase the diversity of genotypes analysed, an isolate from a person infected in Henan province, China (strain CHN_HEN01) was also genotyped. Strain CHN_HEN01 clustered within 1 of the 2 American populations, yet possessed alleles at some markers never observed among American isolates (Nascimento *et al*., 2020; Barratt *et al*., 2021). This finding supported that strain CHN_HEN01 represents a third distinct type, consistent with microsatellite-based genotyping analyses carried out by other investigators on Chinese isolates also collected in Henan province (Li *et al*., 2017).

The 2-type population structure observed among American *C. cayetanensis* isolates (Nascimento *et al*., 2020; Barratt *et al*., 2021, 2022) and the distinctness of strain CHN_HEN01, provide the impetus for the present study. We sought to characterize some of the genetic features driving the 2-type population structure observed among American isolates by defining alleles and/or allelic combinations that are unique to each population. We highlight several genetic differences between the 3 types of *C. cayetanensis* (lineages A, B and C, henceforth). Evidence for a lack of gene flow between the lineages is presented, in support of reproductive isolation and therefore, a species level distinction. Retrospective examination of epidemiologic data for genotyped American *C. cayetanensis* (lineages A and B) revealed associations between lineage membership and the geographic distribution of US infections, in addition to strong temporal associations, supporting an ecological distinction. Finally, given the multiple lines of evidence for speciation within human-infecting *Cyclospora*, we provide an updated taxonomic description of *C. cayetanensis*, and introduce 2 novel species as aetiological agents of human cyclosporiasis: *Cyclospora ashfordi* sp. nov. and *Cyclospora henanensis* sp. nov. (Apicomplexa: Eimeriidae).

## Methods

This study was divided into three key objectives: (1) identification of ‘lineage-defining alleles’, (2) examination of loci extracted from published *C. cayetanensis* genomes and (3) retrospective epidemiological and morphological analyses.

### Identification of ‘lineage-defining’ alleles

As revealed by amplicon deep-sequencing, most *C. cayetanensis* genotypes had multiple alleles at multiple loci, attributed to heterozygous parasites and/or mixed-strain infections. This heterogeneity is characteristic of *C. cayetanensis* infections, owing to sexual reproduction which occurs in the gut of infected human hosts (Barratt *et al*., 2019). Because *C. cayetanensis* is unicellular, our genotypes represent an amalgamation of the many individual parasites comprising an infection. Consequently, some genotypes may represent co-infections with multiple unrelated strains, introducing noise to resultant tree structures upon clustering. Additionally, Barratt's heuristic and Plucinski's Bayesian algorithms include routines that address the issue of missing sequence data, allowing distance computation for isolates with a partial genotype (Barratt *et al*., 2019; Barratt and Sapp, 2020; Nascimento *et al*., 2020). While this affords the benefit that partial genotypes (e.g. due to low parasite load or low specimen volume) need not be excluded from a clustering analysis, this is another source of noise because imputation of missing values becomes increasingly tenuous as the number of loci with missing sequence data increases (Barratt and Sapp, 2020; Jacobson *et al*., 2022).

To simplify the identification of alleles driving the 2-type population structure observed, we excluded genotypes that were incomplete and those likely to be derived from mixed-strain infections (see detailed methods below). This would produce a set of ‘strain-pure’ genotypes resulting in a relatively noise-free tree structure that would more clearly highlight population-level trends. Genotypes in this filtered dataset would also reflect allelic combinations that are more likely to occur within a single *Cyclospora* oocyst, which is the product of a sexual cross. Conversely, this would also highlight allelic combinations that are theoretically possible, but rarely (or never) observed. The existence of theoretical genotypes that are rarely (or never) observed would not be consistent with panmixia (i.e. random mating), and would support a lack of gene flow between subpopulations. This would imply reproductive isolation which is evidence of speciation as defined by the biological species concept (Mallet, 2010; Wang *et al*., 2020).

#### Genotypes

From March 2018 to October 2020, a total of 3459 *C. cayetanensis* genotypes were sequenced from fecal specimens collected from patients who received a diagnosis of cyclosporiasis in the USA or Canada, and from 4 specimens collected before 2018 (Nascimento *et al*., 2020; Barratt *et al*., 2021, 2022). The latter 4 specimens included 2 collected in Mexico (in 2016 and 2017), 1 in Guatemala (in 2018) and 1 collected in 2011 from a person infected in Henan province, China. This library of genotypes included positive control specimens and duplicate genotypes sequenced from the same patient. Duplicates and controls were excluded. For repeat genotypes from the same case-patient, the genotype with the fewest missing markers was retained if the genotypes were otherwise identical. Genotypes were also excluded if they were associated with multiple fecal specimens from the same case-patient but were divergent from one another (e.g. possibly due to patients becoming infected by different strains on separate occasions). In all, 2866 genotypes were retained for subsequent analyses. These genotypes were represented in the form of a haplotype data sheet (HDS) (File S1, Tab A); a condensed format for presenting haplotype data (see Barratt *et al*., 2021) that is required as the direct input for Barratt's heuristic and Plucinski's Bayesian algorithms (Barratt *et al*., 2019; Nascimento *et al*., 2020). Raw reads generated for genotyped specimen are publicly available in the National Center for Biotechnology Information (NCBI) database under BioProject accession number PRJNA578931.

#### Genotype filtering to identify strain-pure isolates with a complete genotype

Genotypes within the HDS were filtered to retain only those with at least 1 full-length sequence (i.e. 1 allele) for all 8 genotyping markers, to exclude those with more than 1 haplotype for either of the 2 Mt loci, and to exclude those with more than 2 haplotypes for any of the 6 Nu loci. This would leave only complete, ‘strain-pure’ genotypes likely possessing allelic combinations that naturally occur in a single oocyst which is the product of a sexual cross. This is based on the current understanding that sporulated *Cyclospora* oocysts possess 2 sporocysts that each contain 2 haploid sporozoites, where twin sporozoites (i.e. those in the same sporocyst) are genetically identical while sporozoites in different sporocysts of the same oocyst may be distinct, such that a single oocyst may be heterozygous (Babiker *et al*., 1994; Ortega *et al*., 1994; Almeria *et al*., 2019).

#### Genetic distance computation and hierarchical clustering of strain-pure genotypes

A genetic distance matrix was computed from the strain-pure HDS using Barratt's heuristic (Nascimento *et al*., 2020), *via* the R code accessible at: https://github.com/Joel-Barratt/Eukaryotyping. Barratt's heuristic is 1 of the 2 algorithms underpinning module 2 (Barratt *et al*., 2021), and the decision to use only Barratt's heuristic (as opposed to both algorithms) was based on a recent evaluation showing that using Barratt's heuristic alone improves clustering accuracy compared to using both algorithms (Jacobson *et al*., 2022). Hierarchical clustering of the resulting distance matrix was carried out using Ward's method (Nascimento *et al*., 2020), and a hierarchical tree was rendered using the ggtree R package (Yu *et al*., 2016).

#### Identification of lineage-defining alleles

The hierarchical tree revealed 2 distinct populations. Based on this observation, the tree was dissected into 2 partitions using the cutree R function, where lineages A and B occupied 1 of the 2 resultant partitions. The genotype of isolates in each partition was examined to identify alleles and/or allelic combinations that are unique to each lineage. Once these lineage-defining alleles were identified, we returned to the unfiltered library (*n* = 2866 genotypes) and assigned these genotypes to either a known lineage, as possessing a mixed-lineage genetic background, or as belonging to an unknown lineage. These designations were made based solely on their possession (or lack) of certain lineage-defining alleles.

### Examination of loci from published *C. cayetanensis* genomes

To expand our analysis beyond 8 markers, we extracted additional loci from published whole-genome shotgun (WGS) sequences, Mt genomes and apicoplast genomes. Each WGS sequence was assigned to lineage A, B or C by BLASTN searches using lineage-defining alleles identified *via* the first objective as query sequences and the genomes as references. Full-identity BLASTN hits to specific lineage-defining alleles would confirm a genomes lineage membership. Loci extracted from WGS sequences of known lineage would facilitate further cross-lineage comparison by phylogenetic analysis to explore whether these loci support a pattern of reciprocal monophyly, which is strong evidence for speciation (de Leon and Nadler, 2010).

#### Examination of housekeeping genes extracted from published genomes

Assignment of 34 published *C. cayetanensis* WGS sequences (Qvarnstrom *et al*., 2015; Liu *et al*., 2016; Qvarnstrom *et al*., 2018; Barratt *et al*., 2019) to lineage A, B or C was achieved by BLASTN searches as discussed above, and hits to various housekeeping loci were extracted from these genomes for phylogenetic comparison. Housekeeping genes are well characterized, highly conserved loci that are usually fundamentally important to cellular functioning (Joshi *et al.*, 2022). These loci are often the focus of phylogenetic and taxonomic studies involving protozoa because their sequence is usually conserved among isolates of the same species (Stensvold *et al*., 2013; Kaufer *et al*., 2017). Sequences of *Cyclospora* sp. 18S rDNA (GenBank: AF111187.1), actin (ToxoDB: cyc_03710-t31_1), lactate dehydrogenase (ToxoDB: cyc_04011-t31_1), RNA polymerase II subunit (ToxoDB: cyc_08603-t31_1) and 2 paralogues of beta-tubulin (GenBank: XM_022730720.2, XM_022732816.1) were used as query sequences in BLASTN searches against the 34 genomes to identify their genomic location and extract their sequence for phylogenetic analysis.

#### Comparison of Mt genomes, apicoplast genomes and other loci

We empirically selected 3 *C. cayetanensis* hypothetical protein genes from the veupath DB (VDB) reference database (https://veupathdb.org/), including the sequences cyc_06176-t31_1, cyc_06177-t31_1 and cyc_06182-t31_1. These were extracted from the 34 WGS sequences as described above. Two additional empirically selected protein-coding loci – a putative cysteine proteinase gene (VDB: cyc_00943) and a partial sequence of a polyamine-modulated factor 1-binding protein 1 (VDB: LOC34622638) – were also extracted for comparison. We extracted complete or partial apicoplast sequences from each WGS sequence using a complete apicoplast genome sequence available in GenBank for strain CHN_HEN01 (accession: NC_028632.1) as a query sequence. Mt genomes were sequenced at CDC by PCR and Sanger sequencing for several isolates from among the same 34 strains with a published WGS sequence including isolates CDC:HCVA02:15, CDC:HCNY16:01, CDC:HCGM11:97, CDC:HCTX69:14 (lineage A – see Table 1), and isolate CDC:HCRI01:97 (lineage B – Table 1). The sequences of these loci/genomes were subjected to phylogenetic analysis. Finally, large segments of the *C. cayetanensis* Nu genome were selected in a semi-random fashion for phylogenetic analysis. Briefly, contigs from one genome assembly for strain CHN_HEN01 (GenBank Assembly Database Accession: ASM289330v1) were sorted from largest to smallest in length. Several contigs were selected at random from the top of this list (i.e. from among the largest contigs) and BLASTed (BLASTN) against the genomes of American *Cyclospora* isolates to identify large homologous sections from within their Nu genomes. Contigs from strain CHN_HEN01 that obtained a match to contigs in these American *Cyclospora* genome assemblies were noted and matching regions were extracted from these American genome assemblies. This process was continued until approximately 1 million homologous bases of the *Cyclospora* Nu genome (around 2% of the whole genome) had been captured. These large sections of the Nu genome were concatenated to produce a single contig of around 1 million nucleotide bases in length for each isolate, for subsequent phylogenetic analysis.

**Table 1.** Lineage designation of 34 published genomes based on BLASTN searches

Strain	BioSample	GB assembly accession	Origin
American (lineage A)^a^			
HCTX542_15	SAMN10261105	GCA_003935065.1	USA, TX
HCDC004_96	SAMN10261092	GCA_003945175.1	USA, DC
HCGM012_97	SAMN10261094	GCA_003945085.1	Guatemala
HCTX495_16	SAMN10261102	GCA_003944975.1	USA, TX
HCTX503_16	SAMN10261103	GCA_003944985.1	USA, TX
CDC:HCNY16:01	SAMN07337742	GCA_002893425.1	USA, NY
CDC:HCTX48:14	SAMN07337743	GCA_002893485.1	USA, TX
CDC:HCFL47:13	SAMN07337744	GCA_002893405.1	USA, FL
CDC:HCVA02:15	SAMN07337745	GCA_002893375.1	USA, VA
CDC:HCTX227:15	SAMN07337750	GCA_002893465.1	USA, TX
CDC:HCGM11:97	SAMN07337753	GCA_002893445.1	Guatemala
CDC:HCGM01:97	SAMN05982179	GCA_002019465.1	Guatemala
CDC:HCTX69:14	SAMN05982170	GCA_002019455.1	USA, TX
CDC:HCNY16:01	SAMN03983352	GCA_001305735.1	USA, NY
American (lineage B)^b^			
HCGM002_97	SAMN10261093	GCA_003945135.1	Guatemala
HCMX010_16	SAMN10261097	GCA_003945065.1	Mexico
HCTX460_16	SAMN10261101	GCA_003944945.1	USA, TX
HCTX535_14	SAMN10261104	GCA_003944965.1	USA, TX
CDC:HCTX205:15	SAMN07337747	GCA_003057635.1	USA, TX
CDC:HCTX204:15	SAMN07337746	GCA_002893365.1	USA, TX
CDC:HCTX230:15	SAMN07337751	GCA_002893315.1	USA, TX
CDC:HCRI01:97	SAMN05982183	GCA_002019905.1	USA, RI
HCNE181_16	SAMN10261098	GCA_003945055.1	USA, NE
Chinese (lineage C)^c^			
CHN_HEN01	SAMN07337752	GCA_002893305.1	China, Henan
CHN_HEN01	SAMN02944928	GCA_000769155.2	China, Henan
Nepalese (lineage A)^a^			
C5	SAMN04870148	GCA_003351285.1	Nepal
C8	SAMN04870149	GCA_003351265.1	Nepal
NF1_C8	SAMN08618443	GCA_002999335.1	Nepal
NF1	SAMN03145675	GCA_003351245.1	Nepal
C10	SAMN04934518	GCA_003351235.1	Nepal
Nepalese (lineage B)^b^			
HCNP016_97	SAMN10261099	GCA_003945145.1	Nepal
Indonesian (lineage A)^a^^,^^d^			
HCJK015_15	SAMN10261096	GCA_003945155.1	Indonesia, Jakarta
HCJK011_15	SAMN10261095	GCA_003945075.1	Indonesia, Jakarta
Indonesian (lineage B)^‡,^^d^			
CDC:HCJK01:14	SAMN05982165	GCA_002019475.1	Indonesia, Jakarta

a BLASTN searches confirmed that these genomes contain A-lineage alleles of 360i2.

b BLASTN searches confirmed that these genomes contain B-lineage alleles of 360i2.

c The 2 published genomes of strain CHN_HEN01 (and the CHN_HEN01 genotype) were generated from the same biological material. The original material was collected from a 67-year-old male patient in Henan province, China by Longxian Zhuang. This sample was donated to CDC for molecular research by Dr Lihua Xiao. For details, refer to the following BioSample numbers provided in the table.

d These isolates were collected from individuals who travelled to Jakarta and returned to the USA where they received a diagnosis of cyclosporiasis. It cannot be excluded that these patients obtained their infections in the USA.

*Note*: Assignment to lineage A or B was based on a full-length or partial BLASTN match to any of the A-lineage or B-lineage haplotypes defined in Fig. 2. The precise location of the 360i2 matches in each genome is provided in File S1.

### Epidemiological and morphological analyses

Most *C. cayetanensis* genotypes analysed in this study were sequenced to complement cyclosporiasis outbreak investigations in the USA and were therefore associated with epidemiologic information (e.g. food consumption histories and demographic data) collected from cyclosporiasis case-patients from whom fecal specimens were processed for genotyping. Following identification of lineage-defining alleles using our ‘strain-pure’ dataset, we returned to the original unfiltered library (i.e. the ‘noisy’ dataset) and assigned these types to a lineage based solely on their possession of certain lineage-defining alleles. Epidemiologic data linked to genotypes in this larger dataset were examined (as opposed to the smaller, strain-pure dataset) to maximize the power of subsequent epidemiologic analyses which sought to investigate epidemiologic differences between lineages A and B that may underpin an ecological distinction. We also carried out a retrospective morphological analysis by comparing the size of unsporulated oocysts from genetically characterized isolates of each lineage.

#### Analysis of epidemiologic data

Epidemiologic analyses considered a subset of genotypes extracted from the unfiltered dataset of 2866 genotypes (File S1, Tab A). To ensure our findings reflect recent US epidemiologic trends, genotypes from isolates collected outside the USA and those collected in the USA before 2018, were excluded leaving only genotypes generated for the US cyclosporiasis peak periods of 2018–2020. Remaining genotypes not linked to epidemiologic information were excluded, in addition to those of a mixed-lineage background, and those that could not be assigned to a lineage due to the lack of a lineage-defining sequence. In all, 1243 genotypes from isolates collected in the USA from 2018 to 2020 remained.

Epidemiologic information for these 1243 genotypes had been collected through Cyclosporiasis National Hypothesis Generating Questionnaires (CNHGQ) during routine public health surveillance in the USA. Each CNHGQ contained information on a case-patient's basic demographics, travel history, clinical illness and food consumption during a 2-week period prior to illness onset. Because exposure due to travel was not well-defined (i.e. length of stay may vary, and case-patients might not have purchased or eaten produce during their visit, or failed to provide this information) and little traceback information was available on the source of produce items, state of residence was chosen as a proxy for the geographical origin of *C. cayetanensis* isolates. Fisher's exact tests were used to assess the statistical significance of differences between the proportions of lineage A isolates and of lineage B isolates from each state. Time of illness onset was transformed into a categorical variable based on month, and temporal differences between the 2 lineages were similarly assessed. *P* values less than 0.05 were considered statistically significant for all epidemiologic analyses.

#### Morphological analysis

Three randomly selected, genetically characterized isolates of lineages A and B, and the single isolate of lineage C, were subjected to morphological analysis. Stool specimens were concentrated using formalin–ethyl acetate sedimentation and wet mounts were examined under differential interference contrast (DIC) and ultraviolet (UV) fluorescence. Length and width of a minimum of 20 oocysts per sample were measured *via* Olympus cellSens V3.2 software and an Olympus BX51 compound microscope by the same operator. Minimum and maximum dimensions from each lineage were compared using unpaired 2-tailed *t*-tests. The appearance of unsporulated oocysts from each lineage was also examined in modified acid-fast (Kinyoun) stained smears (1000×) and/or under a UV fluorescence microscope (500×).

## Results

### Genotype filtering for strain-purity and completeness, and subsequent clustering

**Fig. 1.**
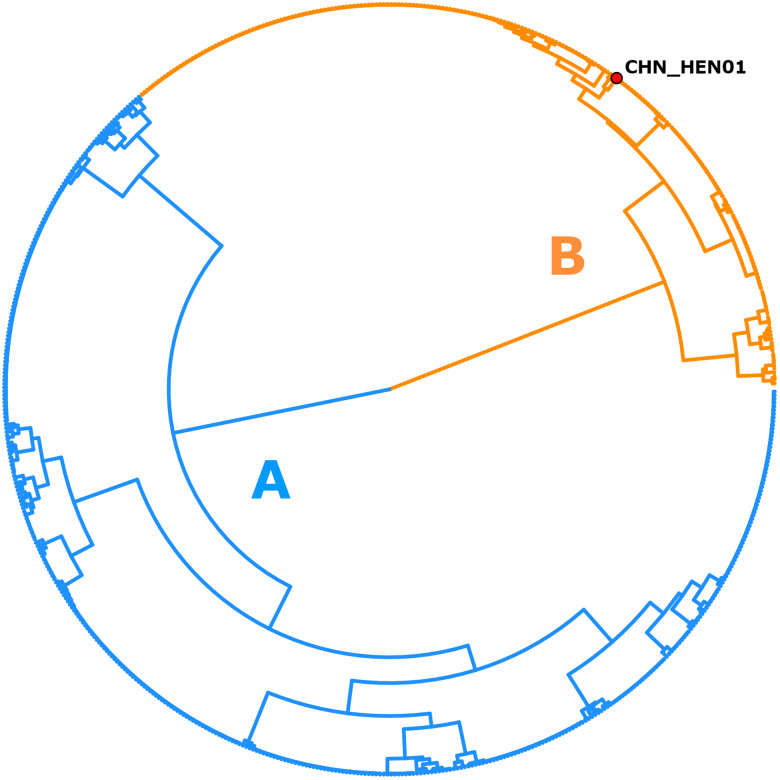
Population structure of *Cyclospora cayetanensis*. This hierarchical tree was generated from a distance matrix computed using Barratt's heuristic definition of genetic distance, including 651 genotypes that had been filtered for strain pureness and genotype completeness. Two distinct populations are supported: lineage A (blue) and lineage B (orange). Strain CHN_HEN01 from Henan, China was clustered alongside 650 American isolates, and its final position within the resultant hierarchical tree supports that it shares some genetic features with lineage B. Despite this, strain CHN_HEN01 clustered as a singleton supporting that it also possesses some unique genetic characters not observed in American isolates.

Following removal of duplicates and controls, 2866 of the original 3459 genotypes remained (File S1, Tab A). After filtering for strain-purity and genotype completeness, 651 genotypes from isolates collected in either the USA, Canada or Mexico remained, in addition to the genotype of strain CHN_HEN01 (File S1, Tab B). Barratt's heuristic (Jacobson *et al*., 2022) was used to compute a distance matrix from these 651 genotypes (File S1, Tab C), and a cluster dendrogram was generated from the resulting matrix revealing 2 distinct populations (Fig. 1). The position of strain CHN_HEN01 within this tree structure supported that it shares some genetic features with lineage B, though it clustered as a singleton supporting that it also possesses some genetic characters not observed in the American isolates (Fig. 1).

### Identification of lineage-defining alleles from the CYCLONE genotyping markers

The genotype of isolates in groups A and B (Fig. 1) was examined for the presence of alleles that were lineage-defining. After dissecting the dendrogram into 2, 416 isolates fell within lineage A (64%) and 235 fell within lineage B (36%). Strain CHN_HEN01 fell within lineage B according to the CYCLONE workflow, though comparison of its genotype to American genotypes revealed that it was distinct. Specifically, strain CHN_HEN01 had unique haplotypes at the Mt junction, MSR and 360i2 loci not observed in American types (File S1, Tab B). These differences between strain CHN_HEN01 and American isolates prompted the examination of additional loci extracted from the genomes of American isolates and strain CHN_HEN01. Comparison of the lineage A and lineage B genotypes (i.e. excluding strain CHN_HEN01) revealed that they are distinguished primarily by their possession of certain allelic combinations at the 360i2 locus.

**Fig. 2.**
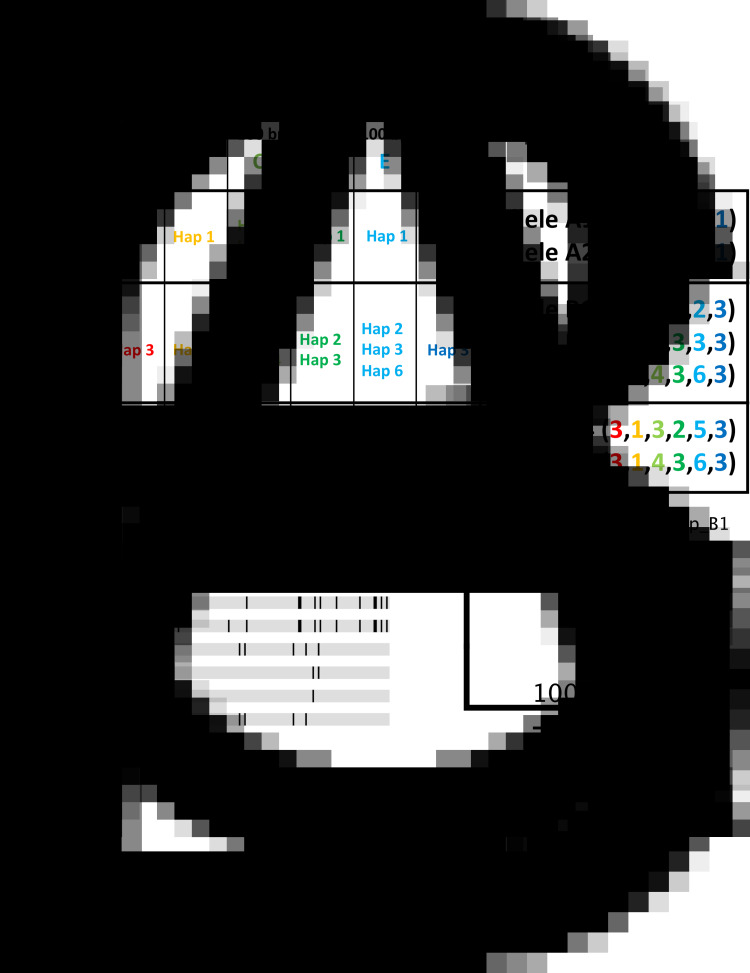
Reconstructing full-length 360i2 alleles and assessing their phylogenetic relationship. The CYCLONE workflow divides the 360i2 amplicon into 6 segments (panel A – segments A–F) and haplotypes are defined separately at each segment. Reconstruction of full-length A-lineage alleles was trivial as a difference only exists at segment C (light green). For lineage B, multiple haplotypes were observed at segments C, D and E. Reconstruction of full-length B-lineage alleles was relatively simple as multiple B-lineage genotypes were homozygous at this locus (Table 2). Full-length alleles were aligned using MUSCLE aligner and a schematic of this alignment was generated using Geneious Prime (panel B). Each allele is shown as a horizontal track, conserved bases are shown in grey and SNPs that differ to the consensus are shown in black. This alignment was used to generate a UPGMA phylogeny (panel C) based on the Jukes–Cantor model, with 1000 bootstrap replicates. Bootstrap percentages are shown on nodes. The scale bar represents numbers of substitutions per site. Panel C shows that haplotypes A1 and A2 share a closer phylogenetic relationship to each other than to the 4 B-type alleles, which form their own distinct clade. The sequence of each haplotype is provided in File S2.

In accordance with CYCLONE workflow (Barratt *et al*., 2021), amplicons of each Nu locus and the MSR locus are divided into segments (i.e. ‘PARTS’ – see loci names in File S1, Tab A) of approximately 100 bases, and haplotypes are defined separately at each segment (Nascimento *et al*., 2020; Barratt *et al*., 2021). The practice of splitting amplicons into sub-segments was adopted to mitigate the impact of PCR-induced chimaeras which could lead to detection of false alleles (Barratt and Sapp, 2020; Nascimento *et al*., 2020). The 360i2 amplicon is 650 bases long, so full-length 360i2 alleles were constructed by concatenating each of 6 distinct segments: A through F (Fig. 2). Briefly, this was achieved by identifying genotypes with a single haplotype at each of its 6 360i2 segments – i.e. homozygous types. Nineteen genotypes met this criterion (Table 2), simplifying reconstruction of all full-length 360i2 alleles. Five full-length 360i2 alleles (610 bases – excluding priming sites) were identified among American isolates, plus a 6th allele unique to strain CHN_HEN01. The 360i2 alleles unique to lineage A, lineage B and strain CHN_HEN01 were defined (Fig. 2A), and assigned a name (alleles A1, A2, B1, B2, B3 and B4). They were aligned (Fig. 2B), and an unweighted pair group method with arithmetic mean (UPGMA) phylogeny was generated (Fig. 2C). For lineages A and B, this 360i2 phylogeny revealed a pattern of reciprocal monophyly (Leliaert *et al*., 2014); A-type alleles (A1 and A2) were phylogenetically closer to each other than to B-type alleles (B1 through B3), which also share a closer relationship to each other than to A-type alleles. However, when considering strain CHN_HEN01 (possessing alleles B3 and B4) 360i2 supported a pattern of paraphyly among the B-type alleles, as lineages B and C shared allele B3 (Fig. 2C).

**Table 2.** Frequency of genotypes observed at the 360i2 locus in the filtered strain-pure population

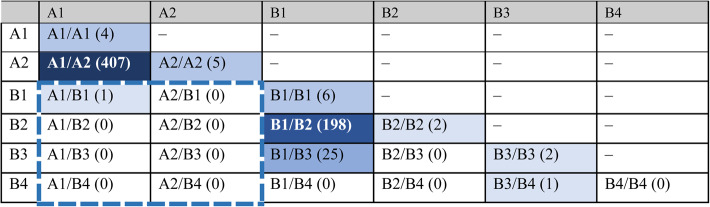

*Note*: This table includes 651 genotypes: 416 from lineage A and 234 from lineage B as defined in Fig. 1. According to the CYCLONE method, strain CHN_HEN01 (with alleles B3 and B4) was also assigned to lineage B. The table also includes a single ‘mixed’ strain with the genotype A1/B1. All 360i2 genotypes (i.e. including theoretical genotypes that assume inter-lineage mixing) are shown and the observed frequency of these genotypes is provided within parentheses. Allelic combinations that were observed in our large dataset are shaded in blue where darker shades reflect higher frequencies and lighter shades reflect lower frequencies. A dashed border surrounds genotypes comprising theoretical mixed-lineage combinations. Only 1 of 8 theoretical A/B allelic combinations was observed comprising a single isolate (0.15% of 651).

Our comparisons also revealed that possession of either of 2 possible CDS3 haplotypes was usually predictive of membership to lineage A or B/C. Briefly, 405 of 416 genotypes (97%) assigned to lineage A (Fig. 1) possessed haplotype 1 of CDS3; only 7 lineage A isolates possessed haplotype 2 (2%), and 4 (1%) possessed both haplotypes 1 and 2. In contrast, 225 of 235 (96%) isolates assigned to lineage B possessed haplotype 2 of CDS3; only 5 lineage B isolates (2%) possessed haplotype 1, and 5 possessed both haplotypes 1 and 2 (File S1, Tab B). Strain CHN_HEN01 was counted among the 225 isolates possessing CDS3 haplotype 2 and so (like 360i2), CDS3 did not support a clear distinction between lineages B and C.

### Frequency of 360i2 allelic combinations for isolates in the strain-pure dataset

One 360i2 allele was unique to strain CHN_HEN01 (allele B4), 2 were exclusive to lineage A (A1 and A2), 2 were exclusive to lineage B (B1 and B2) and 1 was shared between strain CHN_HEN01 (lineage C) and lineage B (allele B3). The frequency of all 360i2 allelic combinations observed among the 651 strain-pure genotypes was tabulated (Table 2) and only 1 isolate (0.15%) possessed a mix of A- and B-lineage alleles (i.e. alleles A1 and B1). Various 360i2 allelic combinations were observed among American isolates. This included ‘homozygous’ types with 1 of A1, A2, B1, B2 or B3. ‘Heterozygous’ types were also observed, including those with A1 and A2, or various combinations of 2 B-type alleles: B1, B2 and B3. In all, 19 ‘homozygous’ types were observed, supporting that the 360i2 locus is encoded once in the haploid *C. cayetanensis* genome (i.e. it is a single-copy locus), and that the ‘heterozygosity’ observed at this locus was not due to multiple paralogous copies of 360i2 in the haploid genome. While infections comprising only A-type or only B-type alleles were extremely common (99.85%), infections comprising A- and B-lineage mixes were exceptionally rare (0.15% of 651) (Table 2). This is despite the high proportion of both A- and B-lineage infections observed in the Americas over multiple years (64 and 36%, respectively). The rarity of these A/B lineage genotype mixes suggests that they represent mixed infections as opposed to infections caused by isolates of mixed-lineage genetic heritage. The patterns observed for CDS3 also support a markedly low frequency of inter-lineage mixing, where 97% of lineage A isolates possess haplotype 1, 96% of lineage B isolates possess haplotype 2 and 1.4% of all isolates (9 of 651) possess both haplotypes.

### Frequency of 360i2 allelic combinations in genotypes from the unfiltered dataset

We next used the 360i2 locus to assign genotypes in the larger unfiltered dataset (*n* = 2866) to a lineage. A total of 483 genotypes lacked a sequence for 360i2 and could not be classified. Of the remaining 2383 genotypes, 65% (*n* = 1550) were assigned to lineage A, 32% (*n* = 752) were assigned to lineage B and 3% (*n* = 80) were an A/B mix, and 1 of the genotypes (<1%) was from strain CHN_HEN01 (lineage C). As shown in File S1 Tab E, most of the 80 mixed-lineage genotypes were highly complex, possessing 3–4 alleles at many of their Nu markers, and sometimes 2 or 3 alleles at their Mt makers. The most likely explanation is that these represent polyinfections, resulting from exposure to multiple strains rather than exposure to single strains that are the product of true A/B lineage sexual crosses.

### Phylogenetic analysis of Mt genomes, apicoplast genomes and other loci

**Fig. 3.**
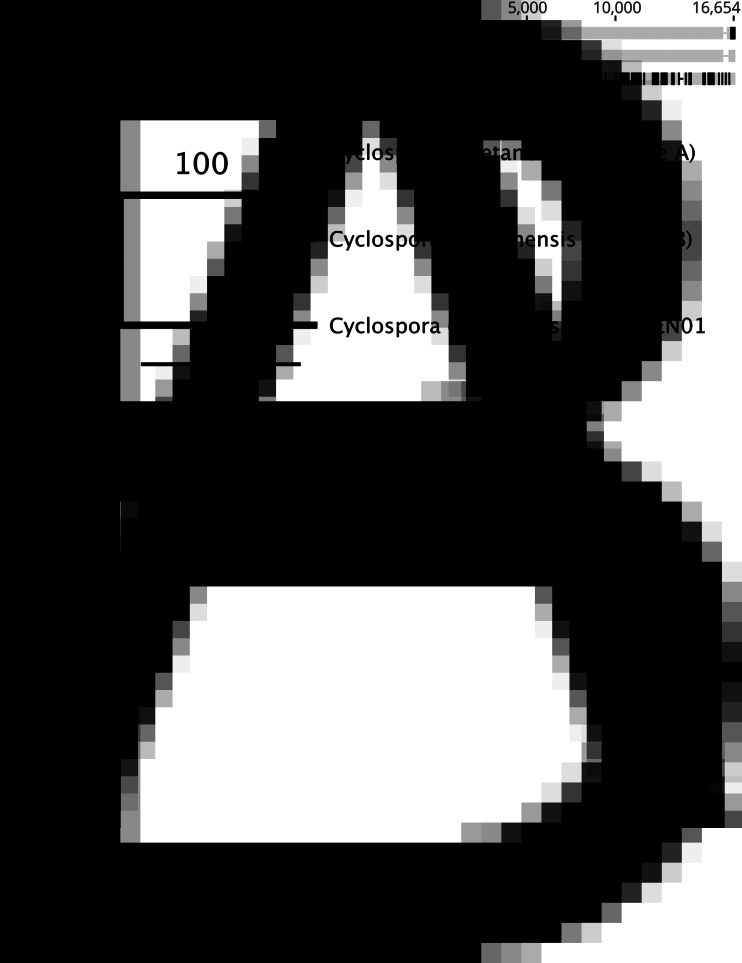
Alignment of concatenated sequences from several loci and the Mt genome (A) and the resulting phylogeny (B). This phylogeny (panel B) was generated by concatenating several loci extracted from published WGS sequences to Mt genomes of representative isolates of lineage A, lineage B and strain CHN_HEN01. The loci were concatenated in the following order to produce the alignment in panel A generated in Geneious Prime: Mt genome excluding the junction (positions 1–6020), beta-tubulin paralogue 1 (6021–6878), cyc_06182-t31_1 (6879–8345), cyc_06177-t31_1 (8346–12 999), cyc_06176-t31_1 (1300–16 446), 360i2 part A (16 447–16 546) and 360i2 part F (16 547–16 656). Sequences were aligned using MUSCLE. Sequences obtained for each lineage are shown as a horizontal track, conserved bases are shown in grey and SNP's that differ to the consensus are shown in black. Gaps are represented by a dash. This alignment was used to generate a UPGMA phylogeny (panel B) based on the Jukes–Cantor model, with 1000 bootstrap replicates. A bootstrap percentage (100%) is shown on the single node, and the scale bar represents the number of substitutions per site.

Analysis of hypothetical proteins cyc_06176-t31_1, cyc_06177-t31_1 and cyc_06182-t31_1 supported that strain CHN_HEN01 is distinct from lineages A and B, and comprises a third lineage (lineage C). Two sequenced genomes of strain CHN_HEN01 (technical sequencing replicates of the same material) are available in the NCBI database and these genomes were identical at these loci, confirming that its distinctness from lineages A and B is not due to sequencing errors. Strain CHN_HEN01 differed from American isolates by more than 40 single-nucleotide polymorphisms (SNPs) and some indels at these loci while American isolates of lineages A and B were largely identical (File S1, Tab D). Strain CHN_HEN01 also had a unique beta-tubulin paralogue 1 allele not observed in lineage A or B, which were identical at this locus (Table 3). Mt genome alignments also supported the distinctness of lineage C from lineages A and B (Table 3, Fig. 3). Mt genomes of lineage A isolates sequenced at CDC were identical to a published sequence from strain C10 (GenBank accession – GB: MG831588.1), so this sequence was used to represent lineage A in our phylogenetic analysis. The Mt genome of strain CDC:HCRI01:97 (lineage B) sequenced at CDC was identical to the published Mt genome of strain ME_14_CL_25 (GB: MN260351.1), so this sequence was selected to represent lineage B. The Mt genome of strain CHN_HEN01 was already publicly available (GB: KP796149.1). Representative Mt genomes of lineages A, B and C were concatenated to respective sequences of beta-tubulin paralogue 1, hypothetical proteins cyc_06176-t31_1, cyc_06177-t31_1 and cyc_06182-t31_1 and segments A and F of 360i2 from these lineages to produce a UPGMA phylogeny (Table 3, Fig. 3). This phylogeny supported that lineages A, B and C are distinct, with lineage C (strain CHN_HEN01) separating as a well-supported outgroup.

**Table 3.** Summary of loci examined from published *Cyclospora cayetanensis* WGS sequences used

Locus	GenBank (GB) or ToxoDB (TDB) accession number^a^	Number of alleles detected	Notes
Loci used to construct the phylogeny in Fig. 3			
Mt genome^c^	Numerous^d^	Numerous	Alignment of 30 Mt genomes (American and Nepalese isolates, and strain CHN_HEN01) revealed several haplotypes. After exclusion of the repetitive Mt junction sequence^c^, most isolates differed by 2 or 3 SNPs while strain CHN_HEN01 differed to all other isolates by ~9 SNPs.
Beta-tubulin – paralogue 1	GB: XM_022730720.2	2	1 allele was detected in both published genomes of strain CHN_HEN01. The other allele was found in all other isolates (lineages A and B).
Hypothetical protein	cyc_06182-t31_1	2	1 allele was detected in both published genomes of strain CHN_HEN01. The other allele was found in all other isolates (lineages A and B).
Hypothetical protein	cyc_06177-t31_1	2	1 allele was detected in both published genomes of strain CHN_HEN01. The other allele was found in all other isolates (lineages A and B).
Hypothetical protein	cyc_06176-t31_1	2	1 allele was detected in both published genomes of strain CHN_HEN01. The other allele was found in all other isolates (lineages A and B).
360i2 (parts A and F)	See Appendices	6	Excluding strain CHN_HEN01, this locus supports a pattern of reciprocal monophyly for lineages A and B. However, strain CHN_HEN01 possesses an allele present in lineage B in addition to an allele not observed among American isolates. This supports a pattern of paraphyly for lineage B and strain CHN_HEN01.
Loci used to construct the phylogeny in Fig. 4			
Apicoplast genome, partial^b^	See File S1, Tab D for GB genomic coordinates	Numerous	Alignment of several apicoplast genomes demonstrated that strain CHN_HEN01 differed from all other isolates by numerous SNPs and indels.
Putative cysteine proteinase	TDB: cyc_00943	2	1 allele was detected in both published genomes of strain CHN_HEN01. The other allele was found in all other isolates (lineages A and B).
Polyamine-modulated factor 1-binding protein 1, partial	TDB: LOC34622638	3	Isolates of lineage A, lineage B and strain CHN_HEN01 possessed a unique allele each (i.e. reciprocal monophyly).
Other loci (not included in phylogenies)			
18S rRNA	GB: AF111187.1	1 (see notes)	Partial 18S sequences were extracted from some genomes. Some SNP differences were observed though no clear correlation between membership to lineage A or B, or strain CHN_HEN01 was observed. The location of these SNPs seems random potentially reflective of sequencing errors.
Putative actin	TDB: cyc_03710-t31_1	2	These alleles differ by 1 SNP. No correlation between possession of 1 allele or the other and membership to lineage A or B, or strain CHN_HEN01.
Lactate dehydrogenase	TDB: cyc_04011-t31_1	3	1 allele was detected in genome HCJK011_15 (Indonesia). A second allele was detected in HCNP016_97 (Nepal). Lineages A and B, and strain CHN_HEN01 all possess the third allele.
RNA polymerase II subunit	TDB: cyc_08603-t31_1	2	No correlation between possession of 1 allele or the other and membership to lineage A or B, or strain CHN_HEN01.
Beta-tubulin – paralogue 2	GB: XM_022732816.1	1	1 allele detected shared across all isolates.

a We did not detect all loci in each WGS sequence. For complete BLASTN results refer to File S1 (Tab D).

b The GB accession number for the apicoplast genome of strain CHN_HEN01 is KP866208.1. GB accession numbers for other apicoplast genomes examined: KX273389.1, KX273387.1, KX273385.1, KX273384.1, KX273383.1, KX273382.1, KX273381.1, KX273380.1, KX189066.1, KX273379.1 and KX273386.1. Apicoplast genomes extracted from WGS sequences of various isolates were also examined – refer to File S1 (Tab D).

c The Mt junction region was excluded as its repetitive sequence aligns poorly.

d The GB accession number for Mt genome of strain CHN_HEN01 is KP796149.1. GB accession numbers for other Mt genomes examined: MN316535.1, MN316534.1, MN260366.1, MN260365.1, MN260364.1, MN260363.1, MN260362.1, MN260361.1, MN260360.1, MN260359.1, MN260358.1, MN260357.1, MN260356.1, MN260355.1, MN260354.1, MN260353.1, MN260352.1, MN260351.1, MN260350.1, MN260349.1, MN260348.1, MN260347.1, MN260346.1, MN260345.1, NC_038230.1, KP231180.1, MG831588.1, MG831587.1, MG831586.1, CM003498.1, KP796149.1 and KP658101.1.

**Fig. 4.**
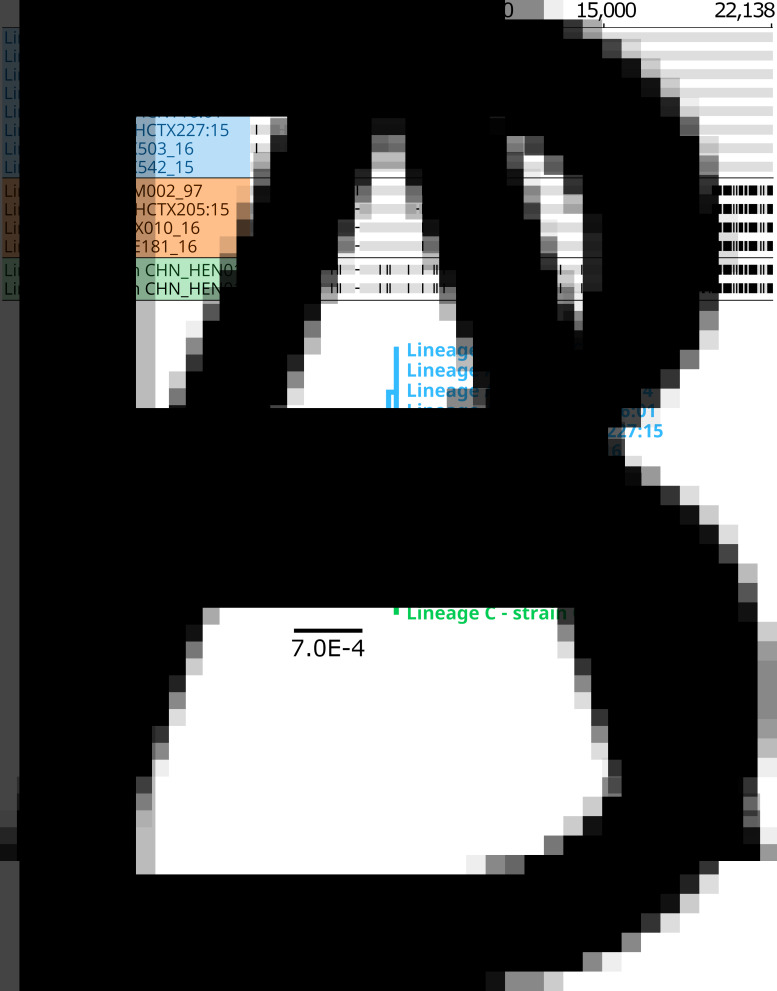
Alignment of concatenated sequences from several loci and partial apicoplast sequences (A) and the resulting phylogeny (B) for several isolates. This phylogeny (panel B) was generated by concatenating 2 protein-coding loci extracted from published *C. cayetanensis* WGS sequences to partial apicoplast genome sequences from the same isolates. These loci were concatenated in the following order to produce the alignment in panel A using Geneious Prime: partial apicoplast genome (positions 1–17 987), putative cysteine protease cyc_00943 (17 988–19 550) and partial sequence of polyamine-modulated factor 1-binding protein 1 – locus LOC34622638 (19 551–22 138). Sequences were aligned using MUSCLE. Each sequence is shown as a horizontal track, conserved bases are shown in grey and SNP's that differ to the consensus are shown in black. Gaps are represented by a dash. This alignment was used to generate a UPGMA phylogeny (panel B) based on the Jukes–Cantor model, with 1000 bootstrap replicates. A bootstrap percentage (100%) is shown on major nodes, and the scale represents the number of substitutions per site. Two sets of sequences obtained for strain CHN_HEN01 were generated from the same material (i.e. technical sequencing replicates – Table 1).

Full-length apicoplast genomes (~34 kb) could not be extracted from some WGS sequences, though a large portion (~18 kb) was recovered from most. The partial apicoplast genomes from American isolates (irrespective of lineage) differed by 3–4 SNPs and a few indels, while the apicoplast genome (i.e. the section analysed) of strain CHN_HEN01 differed from American isolates (irrespective of lineage) by around 28 SNPs and multiple indels. American isolates were identical at the putative cysteine protease gene, while strain CHN_HEN01 possessed a unique allele. A complete sequence of polyamine-modulated factor 1-binding protein 1 (locus LOC34622638) could not be extracted from all genomes, but a comparison of a partial sequence revealed that lineages A, B and C each possess a unique allele (Table 3). The alleles observed in lineages B and C differed by a single SNP while the A-type allele was very distinct. Partial apicoplast genomes (~18 kb) from isolates of lineages A, B and C were concatenated to the cysteine protease gene, and the partial polyamine-modulated factor 1-binding protein 1 sequence to produce a UPGMA phylogeny supporting that the 3 lineages are distinct (Fig. 4).

### Phylogenetic analysis of large segments of the Nu genome

**Fig. 5.**
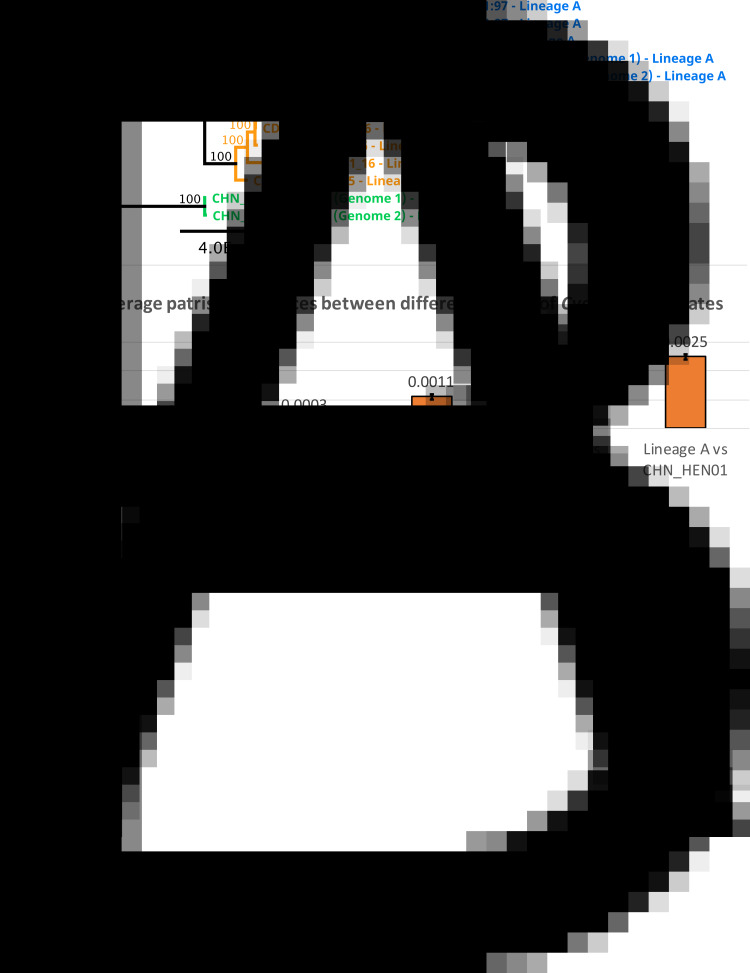
Phylogenetic reconstruction based on large segments of the Nu genome. Several large segments of the *Cyclospora* Nu genome were concatenated for 11 *Cyclospora* isolates. These concatenated genomic segments (~1.02 million bases) were aligned using LASTZ. Genetic distances were computed using the Jukes–Cantor model and a neighbour-joining tree was generated with 1000 bootstrap replicates (panel A). This tree includes 5 isolates assigned to lineages A (blue) and B (orange) based on their possession of A-type or B-type 360i2 alleles, in addition to the 2 sequenced genomes (technical replicates) of strain CHN_HEN01 (green) which cluster as the outgroup. The scale bar represents the number of substitutions per site and bootstrap values are shown on nodes. Two technical (sequencing) replicates of strain CDC:HCNY16:01 included in this analysis are essentially identical supporting the accuracy of these sequences. The precise genomic regions that were concatenated (relative to reference strain CHN_HEN01, GenBank Assembly accession: ASM289330v1) are provided in File S2. Patristic distances were extracted from this phylogeny and average patristic distances between different groups of *Cyclospora* were calculated. Results are represented as a bar chart (panel B). The average distance between isolates of the same lineage is small while the average distance between isolates of different lineages is substantially larger, with the most genetically disparate being lineage C (strain CHN_HEN01). Average patristic distance values are shown above each bar, and error bars represent 1 standard deviation.

A phylogeny generated from 1.02 million nucleotide bases (comprising approximately 2.3% of the *Cyclospora* Nu genome) supported genetic clustering of isolates according to their lineage designation (as shown in Table 1), with strain CHN_HEN01 (lineage C) clustering as a distinct outgroup (Fig. 5). Patristic distances (File S1, Tab G) extracted from the resultant tree structure (Fig. 5) showed that isolates of the same lineage were separated by relatively small distances on average, while the average distance between isolates of different lineages was markedly larger. Notably, the average distance between isolates of lineages A and B was 0.0011 while the distance between the average B-lineage isolate and strain CHN_HEN01, and the average A-lineage isolate and strain CHN_HEN01, was 0.0019 and 0.0025, respectively (Fig. 5); approximately twice the average distance observed between isolates of lineages A and B.

### Analysis of epidemiologic data

**Fig. 6.**
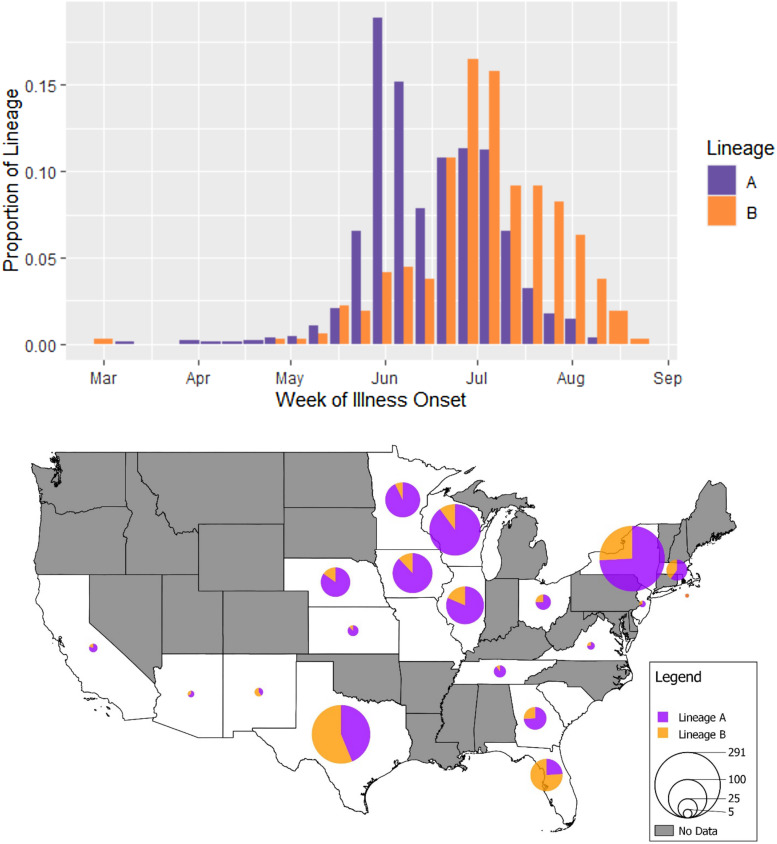
Weekly incidence of cyclosporiasis illness onset and geographical distribution of reported cases attributed to *C. cayetanensis* from lineages A and B over the 2018–2020 cyclosporiasis peak periods. Top (histogram): Dates of illness onset were obtained from CNHGQ data over the 2018–2020 cyclosporiasis peak periods and consolidated into 1 incidence curve by week of illness onset in a side-by-side bar chart. Bar heights indicate the proportions of all cases within a lineage, reporting symptom onsets over a 7-day interval. Proportions are displayed because showing absolute weekly case numbers would mask differences between lineages A (purple) and B (orange) due to the large difference in total numbers of case-patients infected with lineage A *vs* B. Bottom (map): This map shows the distribution of genotyped *C. cayetanensis* isolates from US states reporting cyclosporiasis cases between 2018 and 2020. The pie charts show the relative proportions of each lineage within a state, with lineage A shown in purple and lineage B in orange. No data were obtained from states shaded in grey (i.e. no cases were reported, no specimens were submitted for genotyping or cases/genotypes from these states did not meet the inclusion criteria for this analysis). The size of circles reflects the number of genotyped specimens. Note that no genotyping data were obtained from Alaska or Hawaii.

The 1243 genotypes included in our epidemiologic analysis possessed a relatively balanced distribution of cases across the 3 years (2018: *n* = 322, 2019: *n* = 437, 2020: *n* = 484) and more cases were assigned to lineage A (71.1%, 884/1243) than lineage B (28.9%, 359/1243) overall. Month of illness onset differed significantly between the 2 lineages (*P* < 0.001). Merging 2018–2020 data by time of year, the resulting weekly incidence curve (Fig. 6) reveals a bimodal peak in illness onset for case-patients infected with isolates assigned to lineage A – 1 in the first week of June and the other in the first week of July – compared to a unimodal peak at the first week of July for case-patients infected with isolates assigned to lineage B. Descriptively, a majority of isolates from case-patients with illness onset in the earlier months of April, May and June fall into lineage A (85.7, 84.9 and 83.5%, respectively), and its dominance appears to wane in July (60.2%). In contrast, most isolates from case-patients with illness onset in August were assigned to lineage B (71.0%).

Over the 3 years, there is a clearly observable difference in where isolates from either lineage were more frequently reported. A map (Fig. 6) showing the geographical distribution of lineages A and B reveals that, despite the greater number of lineage A cases overall (more than two-thirds of all reported *C. cayetanensis* cases), lineage B makes up more cases in the southern states of Texas and Florida. A Fisher's exact test comparing geographic distribution between the 2 lineages revealed a statistically significant difference (*P* < 0.001). Narrowing this comparison to individual states, a greater percentage of lineage A isolates originated from cases in states in the Midwest: 10.7% (95/884) of lineage A compared to 3.6% (13/359) of lineage B in Iowa (*P* < 0.001), 8.9% (79/884) compared to 5.0% (18/359) in Illinois (*P* = 0.019), 18.2% (161/884) compared to 5.0% (18/359) in Wisconsin (*P* < 0.001), 8.7% (77/884) compared to 1.7% (6/359) in Minnesota (*P* < 0.001) and 5.9% (50/884) compared to 2.5% (9/359) in Nebraska (*P* = 0.018). Conversely, a greater percentage of lineage B infections occurred in the southern states of Texas and Florida: 36.5% (131/359) of lineage B compared to 11.5% (102/884) of lineage A in Texas (*P* < 0.001), and 15.0% (54/359) compared to 1.9% (17/884) in Florida (*P* < 0.001).

### Morphological comparison

**Fig. 7.**
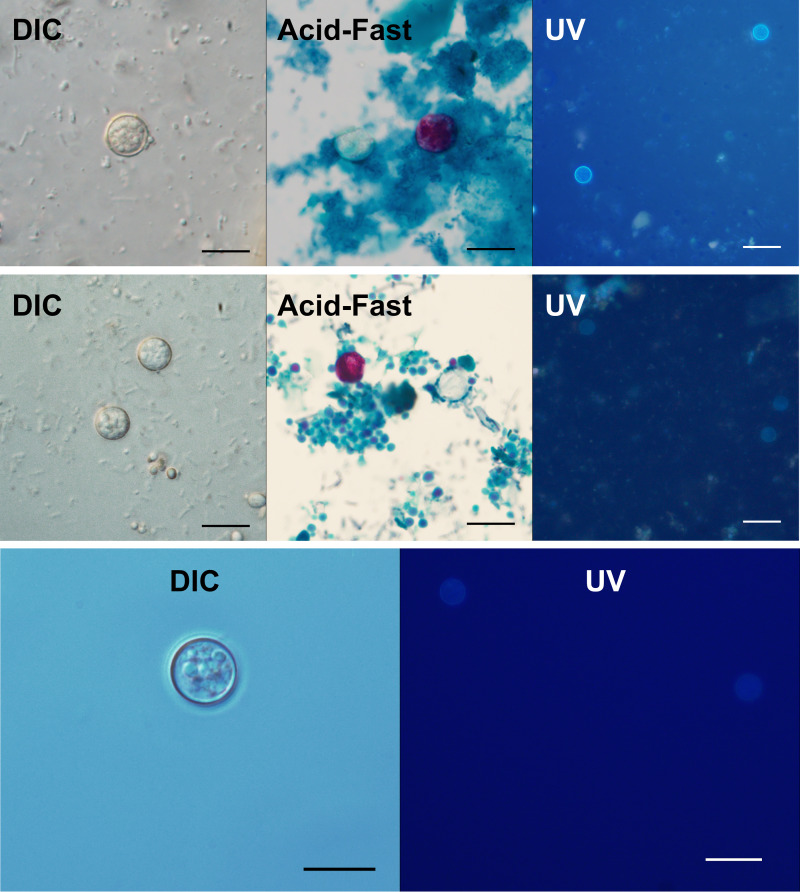
Micrographs of unsporulated oocysts of *C. cayetanensis* lineages A, B and C. Unsporulated oocysts as shown under DIC microscopy (1000×, scale bar 10 *μ*m), on modified acid-fast (Kinyoun) stained smears (1000×, scale bar 10 *μ*m), and under a UV fluorescence microscope (500×, scale bar 20 *μ*m). Top row is lineage A (isolate CTN10041_20), middle row is lineage B (isolate CGA10451_20) and bottom row is lineage C (strain CHN_HEN01). Only UV and DIC images are shown for strain CHN_HEN01 due to limited oocyst material.

Morphometric values are summarized in Table 4. Statistical analysis revealed no significant differences between the minimum and maximum oocyst dimensions when comparing lineage A and B isolates [2-tailed *t*-test; minimum dimension: *P* = 0.5958, 95% confidence interval (CI) −0.1270 to 0.2204; maximum dimension: *P* = 0.6003, 95% CI −0.1979 to 0.1148]. Combined, lineage A and B specimens were found to be slightly smaller than those of strain CHN_HEN01 by about 1 *μ*m in either dimension (2-tailed *t*-test; minimum dimension: *P* < 0.0001, 95% CI −1.2588 to −0.8324; maximum dimension: *P* < 0.0001, 95% CI −1.0487 to −0.6570). One-way analysis of variance (ANOVA) and Tukey's honest significant difference (HSD) also revealed the same pattern of significance for pairwise comparisons (see footnotes in Table 4). Other than the difference in size reported for strain CHN_HEN01 (~10% larger) unsporulated oocysts of the 3 types were similar in appearance (Fig. 7).

**Table 4.** Unsporulated oocyst measurements from each lineage described in this study

	Lineage A	Lineage B	Lineage C
Selected samples	CTN10041_20 CIA10111_20 CGA10281_20	CIL10671_20 CFL10411_20 CGA10451_20	CHN_HEN01
Total number of oocysts measured	79	69	29
Minimum dimension mean (*μ*m) (range)	8.28 (6.59–9.67)	8.24 (6.94–9.41)	9.31^a^ (8.29–10.11)
Maximum dimension mean (*μ*m) (range)	8.61 (7.79–9.93)	8.65 (7.20–9.75)	9.48^b^ (8.35–10.55)

a One-way ANOVA revealed statistical significance among 3 lineages (*F* = 46.79561, *P* < 0.0001). Tukey's HSD was significant between lineage A and strain CHN_HEN01 strain (*P* < 0.0001), and between lineage B and strain CHN_HEN01 (*P* < 0.0001).

b One-way ANOVA revealed statistical significance among 3 lineages (*F* = 36.91703, *P* < 0.0001).

Tukey's HSD significant between lineage A and strain CHN_HEN01 (*P* < 0.0001), and between lineage B and strain CHN_HEN01 (*P* < 0.0001).

## Discussion

In 1979 R. W. Ashford described a novel coccidian encountered during routine fecal examinations carried out in a diagnostic laboratory in Port Moresby, Papua New Guinea (Ashford, 1979). Ashford noted the coccidian's highly regular, spherical oocysts, containing 2 sporocysts, each possessing a number of sporozoites that was difficult to count in the preparations available (Ashford, 1979). Ashford proposed that the oocysts belonged to *Isospora* sensu lato, though admitted they could just as likely be *Toxoplasma* or *Hammondia* (Ashford, 1979; Ortega *et al*., 1994). In 1993, Ortega *et al*. reported the same coccidian in human feces collected in the USA and Peru, following increasing reports of its presence in ‘immunocompetent visitors to developing countries and immunocompromised patients with chronic diarrhea in the United States’ (Ortega *et al*., 1993). A link was noted between these coccidia and reports of ‘cyanobacterium-like bodies’ and ‘*Cryptosporidium muris*-like oocysts’ in the feces of patients with gastrointestinal illness (Ortega *et al*., 1993). These forms were identified as belonging to the genus *Cyclospora* shortly after, based on the morphology of their sporulated oocysts which possessed 2 sporocysts each containing 2 sporozoites; a diagnostic feature of the genus (Ortega *et al*., 1993). In 1994, Ortega *et al*. proposed the name *Cyclospora cayetanensis*; the etymology originating from Cayetano Heredia University, in Lima, Peru, the location where ‘principal studies on this parasite were conducted’ (Ortega *et al*., 1994).

*Cyclospora cayetanensis* has since been recognized as a cause of seasonal gastrointestinal illness in the USA and Canada, having been linked to clusters of illness from as early as 1990 (Soave *et al*., 1998), including several from the late 1990s, such as the 1996 and 1997 outbreaks associated with fresh raspberries imported from Guatemala (Anonymous, 1997; Herwaldt and Ackers, 1997). In 1999 cyclosporiasis became nationally notifiable in the USA (Hall *et al*., 2011), and in 2018, 2299 laboratory-confirmed cases were reported to CDC; the largest number of annually reported cases since the illness became notifiable (Nascimento *et al*., 2020). This was exceeded in 2019, when 2408 cases were reported to CDC (Barratt *et al*., 2021). In 2020, 1441 laboratory-confirmed cases were reported to CDC (Barratt *et al*., 2022), with the reduction from 2019 attributed partly to the impact of the COVID-19 pandemic (Ray *et al*., 2021). Given past trends, *C. cayetanensis* will likely continue to impact US food safety, highlighting the importance of continued investigations into its life cycle, genetics and basic biology.

Given this organism's challenging lack of an animal model or *in vitro* culture system, innovative molecular and bioinformatic approaches are critical for biological investigations. Several recent incremental developments, including development of novel methods for oocyst purification, facilitated sequencing of several *C. cayetanensis* genomes providing information required to identify genotyping markers (Qvarnstrom *et al*., 2015, 2018; Barratt *et al*., 2019; Hofstetter *et al*., 2019; Nascimento *et al*., 2019; Houghton *et al*., 2020). While evaluating these markers, *C. cayetanensis* infections were often observed to be genetically heterogenous, which was attributed to its obligate sexual reproductive cycle (i.e. frequent meiotic recombination events) (Barratt *et al*., 2019). This presented an analysis challenge because traditional alignment-based phylogenetic approaches were not designed to address heterozygosity (Barratt *et al*., 2019; Barratt and Sapp, 2020; Jacobson *et al*., 2022). This motivated the development of our novel genetic distance computation approach (Barratt *et al*., 2019; Nascimento *et al*., 2020; Jacobson *et al*., 2022), and its utilization as an integral part of the CYCLONE *C. cayetanensis* genotyping system (Nascimento *et al*., 2020; Barratt *et al*., 2021, 2022). The utility of this system, particularly the ability to compute genetic distances for heterozygous isolates (Jacobson *et al*., 2022), is pertinent here as the resultant 2-type tree structure (e.g. Fig. 1) highlighted the rarity of certain allelic combinations, supporting a lack of gene flow between the 2 American subpopulations.

Indeed, the lack of gene flow among certain American *C. cayetanensis* isolates evidenced by observed, rarely observed and unobserved, allelic combinations of CDS3 and 360i2 characterizes the distinction between lineages A and B and provides compelling evidence that a pre- or postzygotic barrier exists, preventing sexual reproduction between these lineages. Of CDS3 and 360i2, the various combinations of 360i2 alleles observed provide the strongest support for disrupted gene flow between lineages A and B; among 651 strain-pure genotypes, only 1 possessed a combination of A- and B-type alleles. Notably, examination of the unfiltered genotype library still supported disrupted gene flow between lineages A and B; A/B mixes accounted for around 3% (*n* = 80) of 2383 genotypes that possessed a 360i2 sequence. Lineage A accounts for ~64% of American isolates, and lineage B for ~36%, so neither lineage is rare. Therefore, if the 2 lineages were not reproductively isolated and panmixia (i.e. random mating) were assumed to occur, one would expect far greater than 0.15 or 3% of isolates in the filtered and unfiltered datasets, respectively, to possess a 360i2 A/B combination. Indeed, given these frequencies, and assuming the Hardy–Weinberg principle held true, if a reproductive barrier did not exist and random mating occurred unimpeded, in the F1 generation alone around 23% of infections would represent a mixed A/B type (i.e. 0.64 × 0.36), with approximately 28% of infections being caused by a pure B-type strain, and around 49% being attributed to a pure A-type strain. If random mating continued beyond the F1 generation, subsequent generations would yield far greater numbers of mixed-lineage genotypes such that our clustering analysis (Fig. 1) would not have revealed 2 distinct groups. The pattern observed for CDS3 provides further support for disrupted gene flow between lineages A and B; 97% of genotypes in the filtered dataset with 360i2 alleles A1, A2 or both, possessed only CDS3 haplotype 1 while 96% of isolates with 360i2 alleles B1, B2, B3 or combinations of these, possessed only CDS3 haplotype 2. The strength of these associations excludes coincidence as a possible explanation and reflect patterns of recombination that are inconsistent with panmixia, and therefore provide evidence of speciation (de Leon and Nadler, 2010).

Ultimately, the marked rarity of mixed-lineage infections supports that cross-lineage sexual reproduction rarely (or never) occurs, which is evidence of speciation as defined by the biological species concept (Mallet, 2010; Wang *et al*., 2020). Importantly, multiple 360i2 allelic combinations were observed among the filtered genotypes (e.g. A1/A2, B1/B2, B1/B3, B3/B4), plus the single mixed type (A1/B1), in addition to 19 ‘homozygous’ types. These observations are noteworthy as they support available genomic sequences indicating that the 360i2 locus occurs once in the haploid genome (i.e. it is single copy), such that genotypes possessing 2 alleles reflect the presence of multiple haploid *C. cayetanensis* genome copies within a fecal specimen and not the existence of multiple paralogous 360i2 locus copies within individual haploid genomes. Thus, the ‘heterozygous’ genotypes in our ‘strain-pure’ dataset likely reflect allelic combinations that occur in a single oocyst: the product of a single sexual cross. In further support of a species level distinction, alleles A1 and A2 – seen regularly in combination with one another – are phylogenetically related and are distinct from all B-lineage alleles which are phylogenetically related to each other. This observation of reciprocal monophyly for lineages A and B provides strong support for the distinctness of these lineages (de Leon and Nadler, 2010): alleles frequently observed in combination within the same infection share recent genetic ancestry compared to alleles not frequently observed together.

An investigation into the epidemiologic patterns among lineages A and B is consistent with the genetic observations discussed above. Our analysis of illness onset dates revealed distinct temporal trends between lineages A and B, suggesting that B-type *C. cayetanensis* may be biologically active later in the year compared to A-type *C. cayetanensis*. In aggregate, the difference between their median dates of onset is 13 days (lineage A: 22 June, lineage B: 5 July). While not a huge difference, this may represent quite different exposures if shown to be associated with changes in distribution of food products throughout the year. In addition, the contrasting locations where case-patients infected with these lineages are reported from could be explained by variations in food distribution networks that supply produce to each state or regions of the USA, and the presence of either lineage is likely more closely linked to the farms from which food vehicles are sourced. Epidemiologically defined outbreaks (defined as 2 or more case-patients not in the same household who report the same exposure), however, could have amplified geographic differences between the lineages. These outbreaks are generally contained in 1 geographic region of the USA each year and are predominantly composed of 1 lineage (data not shown). Because they contribute to a large proportion of reported case-patients whose isolates were included in this study (517/1243, 41.6%), they may have an outsized effect on the results. Therefore, comprehensive traceback investigations and food distribution network analyses could help elucidate the causal mechanisms behind these associations and separate sporadic cases from outbreak-related cases. Overall, when taken in consideration of the genetic data presented, these epidemiologic observations represent a hypothesis worthy of continued exploration, acknowledging that collection of data from subsequent years is necessary to reaffirm the strength of these temporal and geographic associations.

The distinctness of strain CHN_HEN01 from lineages A and B was also confirmed here. Strain CHN_HEN01 clustered within lineage B by the CYCLONE method yet it exhibits several unique genetic characters. Firstly, it possesses 360i2 allele B4, which has not been observed among thousands of American isolates to date. The Mt genome of CHN_HEN01 is distinct from that of all American strains; it possesses several unique SNPs and a novel Mt junction sequence. This is noteworthy as the Mt genome is recognized as an important taxonomic marker (Kaufer *et al*., 2019; Schwartz, 2021). Comparisons of apicoplast genome sequences from lineages A and B, and strain CHN_HEN01 also confirmed that strain CHN_HEN01 is different as did our phylogenetic analysis of large segments of the Nu genome (approximately 1.02 million bases). Extraction of patristic distances from this phylogeny (Fig. 5) showed that distances separating strain CHN_HEN01 from the average A- or B-lineage isolate, were approximately double the distance that separates the average A-lineage isolate from the average B-lineage isolate. Strain CHN_HEN01 shared some genetic features with lineage B, including 360i2 allele B3 and CDS3 haplotype 2 which contributed to its clustering within lineage B using the CYCLONE method, albeit as a singleton. Therefore, while the CYCLONE markers detected some unique genetic features in strain CHN_HEN01, these features were insufficient to fully distinguish strain CHN_HEN01 from lineage B upon clustering. Nevertheless, strain CHN_HEN01 was obviously distinct based on our analysis of numerous genetic characters not captured by the current panel of 8 genetic markers, including its apicoplast, Mt and Nu genomes (Figs 3–5). This highlights a limitation of the *C. cayetanensis* CYCLONE method that may warrant the sequencing of additional markers to improve genotyping resolution. In further support of the distinctness of *Cyclospora* types from Henan province generally, using a microsatellite-based method, previous investigators also demonstrated that isolates from Henan province in China are distinct from American isolates (Guo *et al*., 2016; Li *et al*., 2017; Nascimento *et al*., 2019).

## Conclusions

We confirm that at least 3 genetic lineages of *C. cayetanensis* are a cause of human cyclosporiasis. Lineages A and B are responsible for seasonal cyclosporiasis outbreaks in the USA and Canada, while lineage C is represented by a single isolate obtained from Henan province, China. Li *et al.* (2017) published a study supporting the distinctness of strains from Henan province in China using a microsatellite-based genotyping method, and it is possible that the Chinese types described by Li *et al*. might be of the same lineage as strain CHN_HEN01, though this is speculative. The genotyping of additional strains from China and possibly elsewhere in Asia and the Indo-Pacific to explore the diversity and geographic range of C-lineage *C. cayetanensis* is strongly indicated; a limitation of this study is that it included only 1 isolate from this region. Despite this, strain CHN_HEN01 possesses genetic features never observed among many genotyped and genome-sequenced American strains, including distinct apicoplast, Mt and Nu genomes. While we cannot exclude that strain CHN_HEN01 is reproductively compatible with isolates of lineage A or B (or both), the stark absence of various CHN_HEN01-specific alleles among thousands of American types supports reproductive isolation – at least due to a geographic (i.e. a prezygotic) barrier resulting in evolutionary separation by vicariance. For lineages A and B, the mechanisms driving their reproductive isolation are less obvious. The observation of sporadic mixed-lineage infections supports an overlapping geographic origin of A- and B-lineage isolates causing illness in the USA, suggesting that their gametes are unable to produce a viable zygote post-fusion or that their gametes are unable to fuse. If this were not the case, vastly more mixed-lineage infections would be observed: in all, our observations are not consistent with panmixia.

Despite compelling evidence for their reproductive isolation, lineages A and B share many similarities. Several genes including hypothetical protein-coding genes and multiple housekeeping loci were examined. Lineages A and B were often identical at these loci or exhibited multiple alleles that were not predictive of membership to either lineage (i.e. they displayed a pattern of paraphyly). Housekeeping loci are often used to support taxonomic distinctions, and the most widely used housekeeping locus for this purpose is the 18S rDNA; however, reliance on such loci risks underestimating the true species diversity within a taxon (Nadler and De Leon, 2011; Piganeau *et al*., 2011). Given that the 18S rDNA locus is among the most highly conserved DNA segments of life (Isenbarger *et al*., 2008), the idea that using this locus to delimit species might underestimate species diversity should not be completely surprising. Indeed, the present study calls into question the utility of 18S rDNA sequencing for rejecting a hypothesis that subpopulations of related protozoa represent discrete species; we show disrupted gene flow between lineages A, B and C which are largely indistinguishable at this locus. Importantly, this is not the first instance of such an observation in a parasitic protozoan. Consider the apicomplexan parasites *Babesia divergens* and *Babesia* sp. MO1. *Babesia divergens* has a mostly European distribution and possesses a cattle reservoir (Yabsley and Shock, 2013). Alternatively, *B. divergens* has an 18S rDNA sequence that is almost identical to that of *Babesia* sp. MO1, which is biologically distinct from *B. divergens*. *Babesia* sp. MO1 likely has a lagomorph reservoir and has only been found in the USA (Yabsley and Shock, 2013). Also consider the aetiological agents of human African Trypanosomiasis – *Trypanosoma brucei* – which is divided among 3 subspecies; *Trypanosoma brucei rhodesiense*, *Trypanosoma brucei gambiense* and *Trypanosoma brucei brucei*. These subspecies are indistinguishable at their 18S rDNA and are morphologically identical, but differ in their geographic range and certain biological features: *T. b. rhodesiense* causes acute illness and is restricted to East Africa while *T. b. gambiense* causes chronic disease in West Africa (Barratt *et al*., 2010). The third subspecies, *T. b. brucei* is incapable of infecting humans and only infects animals (Barratt *et al*., 2010).

Depending on the loci examined, the phylogenetic relationship between lineages A, B and C shifts. Despite this, our phylogenetic analysis of large segments of the Nu genome (Fig. 5) supports that lineage C is an outgroup to lineages A and B. In any case, a clear phylogenetic signal is rarely observed at the species level particularly among recently diverged species (Leliaert *et al*., 2014; Naciri and Linder, 2015). This may be owing to several factors, including differences in evolutionary rates for different gene families, incomplete fixation of gene lineages and/or incomplete lineage sorting (i.e. deep coalescence) (Leliaert *et al*., 2014). The ‘phylogenetic inconsistency’ of the relationships observed between lineages A, B and C (i.e. specifically whether lineage B is a sister taxon to lineage A or C, which is locus-dependent), may also be due to ancient introgressive hybridization that occurred briefly at the onset of speciation (Nadler and De Leon, 2011). Furthermore, a significant amount of time is required after lineage divergence before reciprocal monophyly becomes apparent (Leliaert *et al*., 2014). Leliaert *et al*. acknowledge that some diagnostic features of speciation such as morphological distinctions, reproductive isolation and reciprocal monophyly (at 1 or more loci) might be apparent for different species, may not arise at the same time or in any given order, or may not arise at all (Leliaert *et al*., 2014). Furthermore, it is increasingly recognized that the biological species concept which necessitates a complete lack of gene flow between distinct species, may be too rigid (Naciri and Linder, 2015; Wang *et al*., 2020). Instead, more recent interpretations pose that related species may be described as separately evolving metapopulations following distinct evolutionary trajectories, with occasional gene flow permissible (Naciri and Linder, 2015; Wang *et al*., 2020). Given what is currently understood on the emergence of novel species, and given the data presented here, we posit that lineages A, B and C are in the nascent stage of speciation, where gene flow is highly disrupted (or absent), in support of reproductive isolation. Reciprocal monophyly was demonstrated for some loci, but not all, suggesting incomplete lineage sorting. Regardless, these 3 lineages possess properties indicating that each is following a distinct evolutionary trajectory (i.e. separate species).

In light of these population-level genetic trends, genetically characterized unsporulated oocysts were re-examined and morphometrics were captured. An unexpected difference between lineage C and lineages A and B was detected, with the former being roughly 1 *μ*m larger on average in either dimension. Although the difference was highly statistically significant, for lineage C the difference represents measurements solely from type strain rather than multiple isolates of this lineage (as was carried out for lineages A and B), and any practical diagnostic utility will be limited due to overlap in size ranges. Nevertheless, given the genetic differences described at length here, the accompanying morphological difference is intriguing and is an example of ‘reciprocal illumination’ that can occur in integrative studies on ‘cryptic species’ (de Leon and Nadler, 2010). A careful examination of additional material, including sporulated material, is a very clear next step, particularly for strain CHN_HEN01.

Our retrospective epidemiologic analysis prompted by the observed genetic differences between lineages A and B represents another example of ‘reciprocal illumination’. Epidemiologic data revealed that lineages A and B cause infections in the USA at different times of the year and tend to concentrate in different regions of the country. This may reflect that the primary sources of foods imported into the USA differ by region at different times of the year, suggesting that prior geographic isolation led to the recent divergence of lineages A and B. The promising results of our early exploration into the epidemiologic relevance of these differences motivate further investigation into their significance from a clinical and public health perspective, for example determining whether particular produce items are more likely to be associated with lineage A or B, and/or whether lineage A or B is more virulent, more transmissible or more likely to cause outbreaks.

Information on the epidemiology and biology of *C. cayetanensis* is increasing. This study leveraged that growing pool of knowledge to demonstrate the distinctness of 3 lineages of *Cyclospora* that infect humans. Our study encompassed comparisons of thousands of isolates sequenced at numerous genes, Mt, apicoplast and Nu genome comparisons, a retrospective epidemiologic investigation and morphological comparisons. In light of the evidence presented, we propose that a species-level taxonomic distinction is appropriate for these 3 (presently) cryptic species. The serendipitous discovery of these cryptic species mirrors the summary provided by Saez and Lozano in 2005 (Saez and Lozano, 2005), when molecular analysis became sophisticated enough to reveal previously hidden diversity:

‘The story repeats itself with increased frequency – a number of individuals belonging to a morphologically recognized species are sequenced (or otherwise genetically characterized), normally at several points (loci) within the genome. Then, often unexpectedly, the various genotypes will cluster in reciprocally monophyletic groups, with no signs of genetic exchange between them’.

In line with the exigent evidence for species status, we conclude the present study with an update to the taxonomic summary of *C. cayetanensis* originally developed by Ortega, Gilman and Sterling (1994). We also introduce 2 novel aetiological agents of human cyclosporiasis: *C. ashfordi* sp. nov. and *C. henanensis* sp. nov., and taxonomic summaries for these novel cryptic species are provided below.

## Taxonomic summaries

### *Cyclospora cayetanensis* (Ortega, Gilman and Sterling, 1994)

*Synonyms: Cyclospora cayetanensis* ‘lineage A’

*Taxonomy:* Phylum Apicomplexa (Levine, 1980), emend. (Adl *et al*., 2005); class Conoidasida Levine, 1988; subclass Coccidia (Leuckart, 1879); order Eucoccidiorida (Léger and Duboscq, 1910); suborder Eimeriorina (Léger, 1911); family Eimeriidae (Minchin, 1903); genus *Cyclospora* (Schneider, 1881).

*Type strains: Cyclospora cayetanensis* strains assigned to lineage A, including HCTX495_16, HCDC004_96, CDC:HCTX69:14, CDC:HCTX48:14, CDC:HCNY16:01, CDC:HCGM11:97, CDC:HCFL47:13 and CDC:HCGM01:97.

*Type host:* Humans, *Homo sapiens.*

*Locality: Cyclospora cayetanensis* causes infections in the Americas though may infect humans elsewhere.

*Morphology:* Detailed morphological descriptions of *C. cayetanensis* have been published previously (Ortega *et al*., 1993, 1997; Almeria *et al*., 2019). Genetically characterized unsporulated oocysts examined in this study measure 6.59–9.67 × 7.79–9.93 *μ*m^2^ (mean 8.27 × 8.61 *μ*m^2^; *n* = 79 oocysts) and are otherwise morphologically consistent with previous descriptions. Stains variably using modified acid-fast (Kinyoun method). No genetically characterized sporulated material or enteric stages were available for examination.

*Etymology: Cyclospora cayetanensis* (lineage A) is a more common cause of infection in the Americas (compared to lineage B) where many isolates included in the original descriptions of *C. cayetanensis* were collected (Ortega *et al*., 1993, 1994). Therefore, we propose that lineage A should retain the original species designation of *C. cayetanensis*, following the original etymology (Winston, 1999).

*Diagnosis: Cyclospora cayetanensis* comprises *Cyclospora* strains assigned to lineage A based on their possession of 360i2 A-type alleles (A1 and/or A2). The following genome-sequenced strains are of American origin, possess 360i2 alleles typical of lineage A and therefore represent isolates of *C. cayetanensis*: HCTX495_16, HCDC004_96, CDC:HCTX69:14, CDC:HCTX48:14, CDC:HCNY16:01, CDC:HCGM11:97, CDC:HCFL47:13 and CDC:HCGM01:97.

### *Cyclospora ashfordi* sp. nov. Barratt, Sapp, Arrowood and Qvarnstrom 2022

*Synonyms: Cyclospora cayetanensis* ‘lineage B’

*Taxonomy:* Phylum Apicomplexa (Levine, 1980), emend. (Adl *et al*., 2005); class Conoidasida Levine, 1988; subclass Coccidia (Leuckart, 1879); order Eucoccidiorida (Léger and Duboscq, 1910); suborder Eimeriorina (Léger, 1911); family Eimeriidae (Minchin, 1903); genus *Cyclospora* (Schneider, 1881).

*Type strains: Cyclospora* strains assigned to lineage B, including HCTX535_14, HCTX460_16, HCNE181_16, HCMX010_16, CDC:HCTX205:15 and HCTX204:15.

*Type host:* Humans, *Homo sapiens.*

*Locality: Cyclospora ashfordi* causes infections in the Americas but may also infect humans elsewhere.

*Morphology:* Genetically characterized unsporulated oocysts measure 6.94–9.41 × 7.20–9.75 *μ*m^2^ (mean 8.24 × 8.65 *μ*m^2^; *n* = 68 oocysts) and are indistinguishable from those of *C. cayetanensis* (Ortega *et al*., 1994). Stains variably using modified acid-fast (Kinyoun method). No genetically characterized sporulated material or enteric stages were available for examination.

*Etymology: Cyclospora ashfordi* was named in honour of R. W. Ashford who first provided a rudimentary description of novel coccidian oocysts during routine fecal examinations carried out in a diagnostic laboratory in Port Moresby, Papua New Guinea. These oocysts were believed to belong to the species *C. cayetanensis*, which was later described in detail by Ortega and colleagues (Ashford, 1979).

*Diagnosis: Cyclospora ashfordi* comprises *Cyclospora* strains of the B lineage, as determined by their possession of B-type 360i2 alleles (B1, B2 and/or B3 – see Appendices). Note that this excludes strain CHN_HEN01 which possesses B-type 360i2 alleles but is distinguished from *C. ashfordi* in the description of *C. henanensis*. The following genome-sequenced strains are of American origin, possess 360i2 alleles typical of lineage B, and therefore represent isolates of *C. ashfordi*: HCTX535_14, HCTX460_16, HCNE181_16, HCMX010_16, CDC:HCTX205:15 and HCTX204:15

### *Cyclospora henanensis* sp. nov. Barratt, Sapp, Arrowood and Qvarnstrom 2022

*Synonyms: Cyclospora cayetanensis* strain CHN_HEN01

*Authority:* Phylum Apicomplexa (Levine, 1980), emend. (Adl *et al*., 2005); class Conoidasida Levine, 1988; subclass Coccidia (Leuckart, 1879); order Eucoccidiorida (Léger and Duboscq, 1910); suborder Eimeriorina (Léger, 1911); family Eimeriidae (Minchin, 1903); genus *Cyclospora* (Schneider, 1881); species (in part) *Cyclospora cayetanensis* (Ortega *et al.*, 1994); species *Cyclospora henanensis* (Barratt, 2022).

*Type strain: Cyclospora* strain CHN_HEN01.

*Type host:* Humans, *Homo sapiens.*

*Locality:* The type strain of *Cyclospora henanensis* was isolated from the stool of *H. sapiens* in Henan province, China, though may infect humans elsewhere.

*Morphology:* Genetically characterized unsporulated oocysts are slightly larger than those of *C. cayetanensis* and *C. ashfordi* sp. nov., measuring 8.29–10.11 × 8.35–10.55 *μ*m^2^ (mean 9.31 × 9.48 *μ*m^2^; *n* = 29 oocysts). All other qualitative features are otherwise morphologically consistent with previous descriptions of *C. cayetanensis* (Ortega *et al*., 1994). No genetically characterized sporulated material or enteric stages were available for examination.

*Etymology: henanensis* ‘from Henan’, reflective of the geographic origin of the type strain.

*Diagnosis:* Strain CHN_HEN01 is the type-strain of *C. henanensis*. It is distinguished from *C. ashfordi* and *C. cayetanensis* based on several genetic loci including its Mt and apicoplast genomes, which are distinct from those of *C. cayetanensis* and *C. ashfordi*.

## Data Availability

All data utilized in this study are publicly available. The genome sequences are published in GenBank and can be located using the accession numbers provided throughout the manuscript. The Illumina data used to generate the MLST genotypes analyzed here are available in the NCBI BioProject database under BioProject number PRJNA578931.

## Supplementary material

The supplementary material for this article can be found at https://doi.org/10.1017/S003118202200172X.
