# Supplementary material for: Cyclospora cayetanensis comprises at least 3 species that cause human cyclosporiasis
Source: Parasitology. Author manuscript; Available in PMC 2023 Apr 25. (PMC10090632; doi:10.1017/S003118202200172X)
Supplement: Supplementary file 1 [file NIHMS1863321-supplement-Supp_file_2.docx]

**Supplementary File S2. Appendices** 

***Cyclospora cayetanensis*** **comprises at least three species that cause human cyclosporiasis**

Joel Leonard Nicholas Barratt*^1^, John Shen^1,2^, Katelyn Houghton^1^, Travis Richins^1,3^, Sarah G. H. Sapp^1^, Vitaliano Cama^1^, Michael J. Arrowood^4^, Anne Straily^1^, Yvonne Qvarnstrom^1^

^1^Parasitic Diseases Branch, Division of Parasitic Diseases and Malaria, Centers for Disease Control and Prevention, Atlanta, GA, 30329, USA

^2^The Rollins School of Public Health, Emory University, Atlanta, GA, USA

^3^Oak Ridge Associated Universities, Oak Ridge, TN, USA

^4^Waterborne Disease Prevention Branch, National Center for Emerging and Zoonotic Infectious Diseases, Centers for Disease Control and Prevention, Atlanta, GA, USA

*Corresponding author:

Joel Barratt: nsk9@cdc.gov

# Appendix A. Fasta sequences of segments A to F of 360i2

>Nu_360i2_PART_A_Hap_1

TTCTTTTCGCACTCTGGCTGCCGCTGCTGGTTCTGCTCAAGCAAAGAGAATGGCACGCGGTCATTTTGGTGTATTTTTCATAATCCGTAGCCTGTGGGGA

>Nu_360i2_PART_A_Hap_3

TTCTTTTCGCACTGTGGCTGCCGCTGCTGGGTCCGCTCAAGCAAAGAGAATGGCACGCAGTCATTTTGGTGTATTTTTCATAATCCGTAGCTTGTGGGGA

>Nu_360i2_PART_B_Hap_1

GGGAATGTCACCTAATGTTGCGTCACTAGCATCGGATGGCAGGAAGGGATTAGGCTCAGATTCGGGCAGCATAAGCTGCAGGAGTTGTAAGGCGGCGGCT

>Nu_360i2_PART_C_Hap_1

GGGCTTCTTGCTGATGTATCGTCGTCTTCTGCCCCATCCACAGATTGCATCTTCTCGGATTAGTAAGTTAGGAGAGCAACGATCCGAGCACATTTAGGAG

>Nu_360i2_PART_C_Hap_2

GGGCTTCTTGCTGACGTATCGTCGTCTTCTGCCCCATCCACAGATTGCATCTTCTCGGATTAGTAAGTTAGGAGAGCAACGATCCGAGCACATTTAGGAG

>Nu_360i2_PART_C_Hap_3

GGGCTTCTTGCTGATGTATCGTCGTCTTCTGCCCCATCCACGGATTGCATCTTCTCAGATAAGTAAGTTAGGAGAGCAACGATCCGAGCACATTTAGGAG

>Nu_360i2_PART_C_Hap_4

GGGCTTCTTGCTGATGTATCGTCGTCTTCTGCCCCATCCACAGATTGCATCTTCTCGGATAAGTAAGTTAGGAGAGCAACGATCCGAGCACATTTAGGAG

>Nu_360i2_PART_D_Hap_1

TAGCTTTCTGGTAGTGCGTCGCAAGAATTTTGCACCATCCTTGCGGGTGCGTAAAAGCAAAGGGTATGCCAAGAGGGCCTCAATTTTTTCGAGTCTGGCT

>Nu_360i2_PART_D_Hap_2

TAGCTTTCTGGTAGTGCGTCGCAAGAATTTTGCACCATCCTTGCGGGTGCGTAAAAGCAAAGGGTATGCCAAGAGAGCCTCAATTTTTCTGAGTCTGGCT

>Nu_360i2_PART_D_Hap_3

TAGCTTTCTGGTAGTGCGTCGCAAGAATTTTGCACCATCCTTGCGGGTGCGTAAAAGCAAAGGGTATGCCAAGAGGGCCTCAATTTTTCTGAGTCTGGCT

>Nu_360i2_PART_E_Hap_1

TGATTCCTGCATGCAAAACGCCTAATCCTAGGAACACACCTTTTCTCATCAAAACTCAACGGTTGCGTGACATCAGATAGCCCTGCTTCTCCCGCAACAG

>Nu_360i2_PART_E_Hap_2

TGATTTCTGCATGCAAAACGCCTAATCCCAGGAACACGCCTGTTCTCATCAAAACTCAACGGTTGCGTGACATCAGATAGCGCTGCTTCTCCCGCAACAG

>Nu_360i2_PART_E_Hap_3

TGATTCCTGCATGCAAAACGCCTTATCCCAGGAACACGCCTGTTCTCATCAAAACTCAACGGTTGCGTGACATCAGATAGCGCTGCTTCTCCCGCAACAG

>Nu_360i2_PART_E_Hap_6

TGATTCCTGCATGCAAAACGCCTTATCCCAGGAACACACCTGTTCTCATCAAAACTCAACGGTTGCGTGACATCAGATAGCGCTGCTTCTCCCGCAACAG

>Nu_360i2_PART_E_Hap_5

TGATTTCTGCATGCAAAACGCCTAATCCCAGGAACACACCTGTTCTCATCAAAACTCAACGGTTGCGTGACATCAGATAGCGCTGCTTCTCCCGCAACAG

>Nu_360i2_PART_F_Hap_1

CTGCAGCGCACGAACAAAGTGCTGTACGCTCTCTTAGAGCGTTCCGATTAAAGATTTAGGGGCAGGCCATGAAGGCGCCAATGCAGCCGTTTTAACAGACCTATGAAATT

>Nu_360i2_PART_F_Hap_3

CTGCAGCGCACGAACAAAGTGCTGTACGCTCTCTTACAACGTTCCGATTAAAGATTTAGGGGCAGGCCATGAAGACACCAATGCAGCCGTTTCAACAGACCTATAAAATT

# Appendix B. Fasta sequences of 360i2 haplotypes

>360i2_Hap_A1

TTCTTTTCGCACTCTGGCTGCCGCTGCTGGTTCTGCTCAAGCAAAGAGAATGGCACGCGGTCATTTTGGTGTATTTTTCATAATCCGTAGCCTGTGGGGAGGGAATGTCACCTAATGTTGCGTCACTAGCATCGGATGGCAGGAAGGGATTAGGCTCAGATTCGGGCAGCATAAGCTGCAGGAGTTGTAAGGCGGCGGCTGGGCTTCTTGCTGATGTATCGTCGTCTTCTGCCCCATCCACAGATTGCATCTTCTCGGATTAGTAAGTTAGGAGAGCAACGATCCGAGCACATTTAGGAGTAGCTTTCTGGTAGTGCGTCGCAAGAATTTTGCACCATCCTTGCGGGTGCGTAAAAGCAAAGGGTATGCCAAGAGGGCCTCAATTTTTTCGAGTCTGGCTTGATTCCTGCATGCAAAACGCCTAATCCTAGGAACACACCTTTTCTCATCAAAACTCAACGGTTGCGTGACATCAGATAGCCCTGCTTCTCCCGCAACAGCTGCAGCGCACGAACAAAGTGCTGTACGCTCTCTTAGAGCGTTCCGATTAAAGATTTAGGGGCAGGCCATGAAGGCGCCAATGCAGCCGTTTTAACAGACCTATGAAATT

>360i2_Hap_A2

TTCTTTTCGCACTCTGGCTGCCGCTGCTGGTTCTGCTCAAGCAAAGAGAATGGCACGCGGTCATTTTGGTGTATTTTTCATAATCCGTAGCCTGTGGGGAGGGAATGTCACCTAATGTTGCGTCACTAGCATCGGATGGCAGGAAGGGATTAGGCTCAGATTCGGGCAGCATAAGCTGCAGGAGTTGTAAGGCGGCGGCTGGGCTTCTTGCTGACGTATCGTCGTCTTCTGCCCCATCCACAGATTGCATCTTCTCGGATTAGTAAGTTAGGAGAGCAACGATCCGAGCACATTTAGGAGTAGCTTTCTGGTAGTGCGTCGCAAGAATTTTGCACCATCCTTGCGGGTGCGTAAAAGCAAAGGGTATGCCAAGAGGGCCTCAATTTTTTCGAGTCTGGCTTGATTCCTGCATGCAAAACGCCTAATCCTAGGAACACACCTTTTCTCATCAAAACTCAACGGTTGCGTGACATCAGATAGCCCTGCTTCTCCCGCAACAGCTGCAGCGCACGAACAAAGTGCTGTACGCTCTCTTAGAGCGTTCCGATTAAAGATTTAGGGGCAGGCCATGAAGGCGCCAATGCAGCCGTTTTAACAGACCTATGAAATT

>360i2_Hap_B1

TTCTTTTCGCACTGTGGCTGCCGCTGCTGGGTCCGCTCAAGCAAAGAGAATGGCACGCAGTCATTTTGGTGTATTTTTCATAATCCGTAGCTTGTGGGGAGGGAATGTCACCTAATGTTGCGTCACTAGCATCGGATGGCAGGAAGGGATTAGGCTCAGATTCGGGCAGCATAAGCTGCAGGAGTTGTAAGGCGGCGGCTGGGCTTCTTGCTGATGTATCGTCGTCTTCTGCCCCATCCACGGATTGCATCTTCTCAGATAAGTAAGTTAGGAGAGCAACGATCCGAGCACATTTAGGAGTAGCTTTCTGGTAGTGCGTCGCAAGAATTTTGCACCATCCTTGCGGGTGCGTAAAAGCAAAGGGTATGCCAAGAGAGCCTCAATTTTTCTGAGTCTGGCTTGATTTCTGCATGCAAAACGCCTAATCCCAGGAACACGCCTGTTCTCATCAAAACTCAACGGTTGCGTGACATCAGATAGCGCTGCTTCTCCCGCAACAGCTGCAGCGCACGAACAAAGTGCTGTACGCTCTCTTACAACGTTCCGATTAAAGATTTAGGGGCAGGCCATGAAGACACCAATGCAGCCGTTTCAACAGACCTATAAAATT

>360i2_Hap_B2

TTCTTTTCGCACTGTGGCTGCCGCTGCTGGGTCCGCTCAAGCAAAGAGAATGGCACGCAGTCATTTTGGTGTATTTTTCATAATCCGTAGCTTGTGGGGAGGGAATGTCACCTAATGTTGCGTCACTAGCATCGGATGGCAGGAAGGGATTAGGCTCAGATTCGGGCAGCATAAGCTGCAGGAGTTGTAAGGCGGCGGCTGGGCTTCTTGCTGATGTATCGTCGTCTTCTGCCCCATCCACAGATTGCATCTTCTCGGATAAGTAAGTTAGGAGAGCAACGATCCGAGCACATTTAGGAGTAGCTTTCTGGTAGTGCGTCGCAAGAATTTTGCACCATCCTTGCGGGTGCGTAAAAGCAAAGGGTATGCCAAGAGGGCCTCAATTTTTCTGAGTCTGGCTTGATTCCTGCATGCAAAACGCCTTATCCCAGGAACACGCCTGTTCTCATCAAAACTCAACGGTTGCGTGACATCAGATAGCGCTGCTTCTCCCGCAACAGCTGCAGCGCACGAACAAAGTGCTGTACGCTCTCTTACAACGTTCCGATTAAAGATTTAGGGGCAGGCCATGAAGACACCAATGCAGCCGTTTCAACAGACCTATAAAATT

>360i2_Hap_B3

TTCTTTTCGCACTGTGGCTGCCGCTGCTGGGTCCGCTCAAGCAAAGAGAATGGCACGCAGTCATTTTGGTGTATTTTTCATAATCCGTAGCTTGTGGGGAGGGAATGTCACCTAATGTTGCGTCACTAGCATCGGATGGCAGGAAGGGATTAGGCTCAGATTCGGGCAGCATAAGCTGCAGGAGTTGTAAGGCGGCGGCTGGGCTTCTTGCTGATGTATCGTCGTCTTCTGCCCCATCCACAGATTGCATCTTCTCGGATAAGTAAGTTAGGAGAGCAACGATCCGAGCACATTTAGGAGTAGCTTTCTGGTAGTGCGTCGCAAGAATTTTGCACCATCCTTGCGGGTGCGTAAAAGCAAAGGGTATGCCAAGAGGGCCTCAATTTTTCTGAGTCTGGCTTGATTCCTGCATGCAAAACGCCTTATCCCAGGAACACACCTGTTCTCATCAAAACTCAACGGTTGCGTGACATCAGATAGCGCTGCTTCTCCCGCAACAGCTGCAGCGCACGAACAAAGTGCTGTACGCTCTCTTACAACGTTCCGATTAAAGATTTAGGGGCAGGCCATGAAGACACCAATGCAGCCGTTTCAACAGACCTATAAAATT

>360i2_Hap_B4_CHN_HEN01

TTCTTTTCGCACTGTGGCTGCCGCTGCTGGGTCCGCTCAAGCAAAGAGAATGGCACGCAGTCATTTTGGTGTATTTTTCATAATCCGTAGCTTGTGGGGAGGGAATGTCACCTAATGTTGCGTCACTAGCATCGGATGGCAGGAAGGGATTAGGCTCAGATTCGGGCAGCATAAGCTGCAGGAGTTGTAAGGCGGCGGCTGGGCTTCTTGCTGATGTATCGTCGTCTTCTGCCCCATCCACGGATTGCATCTTCTCAGATAAGTAAGTTAGGAGAGCAACGATCCGAGCACATTTAGGAGTAGCTTTCTGGTAGTGCGTCGCAAGAATTTTGCACCATCCTTGCGGGTGCGTAAAAGCAAAGGGTATGCCAAGAGAGCCTCAATTTTTCTGAGTCTGGCTTGATTTCTGCATGCAAAACGCCTAATCCCAGGAACACACCTGTTCTCATCAAAACTCAACGGTTGCGTGACATCAGATAGCGCTGCTTCTCCCGCAACAGCTGCAGCGCACGAACAAAGTGCTGTACGCTCTCTTACAACGTTCCGATTAAAGATTTAGGGGCAGGCCATGAAGACACCAATGCAGCCGTTTCAACAGACCTATAAAATT

# Appendix C. Fasta sequences of CDS3 haplotypes

>Nu_CDS3_PART_A_Hap_1

GTTGCTTTATCTTTGGCAGTTAGACAGGGAGGGATTCCTACATTTGCAGGTGATGCTGCTACCTCCATGCGCAGCTGCAACACCAGCAA

>Nu_CDS3_PART_A_Hap_2

GTTGCTTTATCTTTGGCAGTTAGACAGGGAGGGATTCCTACATTTGCAGGTGATGCTGCTACCTCCGTGCGCAGCTGCAACACCAGCAA

# Appendix D. Genomic regions included in the phylogeny used to generate Figure 5

The precise genomic regions (totaling approximately 1.02 million bases) that were concatenated to produce the phylogeny in Figure 5 are listed here (in order of concatenation, and relative to reference strain CHN_HEN01, genome GCA_002893305.1).The genomic coordinates (base pairs) shown in brackets beside each contig number from this genome assembly: PDMJ01000002 (448937:459881), PDMJ01000015 (17542:43229), PDMJ01000015 (140982:175641), PDMJ01000015 (294195:323596), PDMJ01000015 (443184:471814), PDMJ01000005 (257490:346631), PDMJ01000008 (362136:439698), PDMJ01000034 (291283:314071), PDMJ01000034 (278795:287044), PDMJ01000144 (19567:44501), PDMJ01000012 (301373:355044), PDMJ01000036 (15309:47919), PDMJ01000016 (35325:103955), PDMJ01000014 (457019:504389), PDMJ01000014 (185206:193901), PDMJ01000036 (236406:287628), PDMJ01000017 (403948:445461), PDMJ01000017 (66924:99332), PDMJ01000018 (164135:221409), PDMJ01000011 (184746:204377), PDMJ01000009 (478918:509439), PDMJ01000009 (412374:432574), PDMJ01000024 (278708:321758), PDMJ01000024 (57541:109267), PDMJ01000009 (192181:218193), PDMJ01000010 (333758:419709).
